# Simultaneous hyperaccumulation of nickel and cobalt in the tree *Glochidion* cf. *sericeum* (Phyllanthaceae): elemental distribution and chemical speciation

**DOI:** 10.1038/s41598-018-26891-7

**Published:** 2018-06-26

**Authors:** Antony van der Ent, Rachel Mak, Martin D. de Jonge, Hugh H. Harris

**Affiliations:** 1 Centre for Mined Land Rehabilitation, Sustainable Minerals Institute, The University of Queensland, St Lucia QLD, Australia; 2 Laboratoire Sols et Environnement, Université de Lorraine, Nancy, France; 3 Department of Chemistry, University of Sydney, Camperdown, Australia; 4 Australian Synchrotron, ANSTO, Clayton VIC, Australia; 5 Department of Chemistry, The University of Adelaide, Adelaide, Australia

## Abstract

Hyperaccumulation is generally highly specific for a single element, for example nickel (Ni). The recently-discovered hyperaccumulator *Glochidion* cf. *sericeum* (Phyllanthaceae) from Malaysia is unusual in that it simultaneously accumulates nickel and cobalt (Co) with up to 1500 μg g^−1^ foliar of both elements. We set out to determine whether distribution and associated ligands for Ni and Co complexation differ in this species. We postulated that Co hyperaccumulation coincides with Ni hyperaccumulation operating on similar physiological pathways. However, the ostensibly lower tolerance for Co at the cellular level results in the exudation of Co on the leaf surface in the form of lesions. The formation of such lesions is akin to phytotoxicity responses described for manganese (Mn). Hence, in contrast to Ni, which is stored principally inside the foliar epidermal cells, the accumulation response to Co consists of an extracellular mechanism. The chemical speciation of Ni and Co, in terms of the coordinating ligands involved and principal oxidation state, is similar and associated with carboxylic acids (citrate for Ni and tartrate or malate for Co) and the hydrated metal ion. Some oxidation to Co^3+^, presumably on the surface of leaves after exudation, was observed.

## Introduction

Hyperaccumulators are rare plants that accumulate metal and metalloid elements to extraordinarily high concentrations in their living biomass that may be hundreds or thousands of times greater than is normal for most plants^1–3^. Hyperaccumulator plants can achieve such extreme levels of foliar sequestration due to enhanced uptake and translocation mechanisms^4,5^. The hyperaccumulation phenomenon is extremely rare (exhibited by <0.2% of angiosperms) with ~70% of the 700 known hyperaccumulator species recorded for Ni^1,6,7^. Hyperaccumulator plants are found on all continents except Antarctica, in temperate and tropical biomes, with the greatest numbers found in New Caledonia, Cuba and the Mediterranean Region^2,8–10^. The Ni concentrations that hyperaccumulator plants can attain in their leaves and transport fluids can be extreme. For example, two species from Malaysia can accumulate up to 2.4 Wt% Ni in the leaves (*Psychotria sarmentosa* – Rubiaceae) and up to 16.9 Wt% Ni in the phloem sap (*Phyllanthus balgooyi* – Phyllanthaceae) respectively^11,12^.

Hyperaccumulation is not just an interesting biological phenomenon, but holds much promise for evolutionary, genetic and ecophysiological research, and, at the more ambitious end of beneficial use, the potential utilization in phytomining^13^. Nickel phytomining (also termed ‘agromining’) is a special type of farming of hyperaccumulator plants on ultramafic soils, followed by harvesting and incineration of the biomass to produce a high-grade ‘bio-ore’ from which Ni metal or pure salts can be recovered^13^. The criteria for the selection of ‘metal crops’ include high biomass yield combined with high Ni concentrations in the above-ground biomass. Although substantial unrealized opportunities exist in tropical regions for Ni agromining^14^, appropriate agronomic systems have not been developed to date^15^. In temperate and Mediterranean climate regions, phytomining trials using *Alyssum* spp. (Brassicaceae) have yielded >100 kg Ni ha^−1^ per harvest^16^. Considering a nickel price of USD $15 kg^−1^ (average 2010–2016 London Metal Exchange value of Ni) and a potential yield of ≥100 kg Ni ha yr^−1^, the economic potential of implemented Ni phytomining technology is substantial. The commercial returns from phytomining, however, will be finite due to the diminishing concentrations of the target metal in the substrate, but the time scale for economic phytomining may be considerable, estimated at 30 years at least^13^. Cobalt phytomining is potentially more profitable because of its higher value compared to Ni (Ni is $13 kg^−1^ and Co USD $94 kg^−1^ in March 2018), but foliar Co accumulation is much lower than for Ni. The highest foliar Co concentrations have been reported in *Haumaniastrum robertii* (Lamiaceae) from the Democratic Republic of Congo (D. R. Congo) with 1 Wt% or 0.7 Wt% Co in culture^17^. High foliar Co have also been reported in *Rinorea javanica* (Violaceae) with up to 670 μg g^−1^ in natural conditions^18^ and *Alyssum troodii* (Brassicaceae) with up to 2325 μg g^−1^ in spiked soils^19^. In total 32 Co hyperaccumulator plants are known globally, of which 16 are from the south-eastern D. R. Congo growing on highly Co-enriched soils (the so-called ‘Copper Hills’)^20,21^.

Although Co is not essential for plants, Co is required by symbiotic rhizobia of leguminous plants and free-living nitrogen-fixing bacteria^22^, while Ni is part of the enzyme urease^23^. The toxicity of Ni and Co to plants is linked to oxidative stress, inhibition of photosynthesis, and Fe deficiency resulting in retardation and inhibition of growth and chlorosis and necrosis of leaves^24–27^. Hyperaccumulation of Co was first defined as >1000 μg g^−1^ foliar Co^28^, but later revised downwards to >300 μg g^−1 ^^2,3^. Hyperaccumulation essentially consists of two discrete stages, abnormal uptake in the root with enhanced translocation, followed by effective tissue and cell-level sequestration. Nickel hyperaccumulator plants achieve extraordinary levels of specificity for Ni over other transition group elements, such as iron (Fe), Mn and Co as a result of yet unidentified Ni-specific membrane-transporters. Knowledge of the uptake, biotransformation and distribution of Ni in hyperaccumulator plants is critical in understanding the process of metal acquisition and metal tolerance^29^. Most chemical speciation studies on hyperaccumulator plants have reported results from bulk measurements, and therefore could not distinguish between the different compartments across the biopathways, including root, xylem, phloem, and leaves^30^. X-ray absorption spectroscopy (XAS) has been used to study Ni chemical speciation in *Alyssum, Leptoplax* and *Noccaea* hyperaccumulators and mixtures of citrate and malate ligation were reported, which were found to vary in different parts of the plants^31,32^. Limited research to date has applied synchrotron radiation study to tropical Ni hyperaccumulator plants and very little is known about the transformation of Ni species from uptake to storage in plant leaves. Co-localization of multiple elements is suggestive of a common transport system, whereas differential accumulation patterns are suggestive of separate transporter pathways. Recent investigations on the distribution and chemical speciation of Ni in three different hyperaccumulator species from Sabah revealed that Ni is present in the form of Ni:citrate and preferentially accumulated in the epidermis and in the spongy mesophyll in the leaves and in the phloem in the roots and branches^33^.

The recently discovered *Glochidion* cf. *sericeum* (Phyllanthaceae) from Malaysia is unusual in simultaneously hyperaccumulating Ni and Co to approximately 1500 μg g^−1^ in the leaves, *i.e*. a ratio of ~1:1 Ni:Co^11^. Compared to most other Ni hyperaccumulator plants, with a mean Ni:Co of 475 for 24 different species^34^, this response, and the very high foliar Co concentrations it attains, is remarkable. The current study used synchrotron X-ray fluorescence microscopy (XFM) and XAS to investigate whether the distribution and associated ligands for Ni and Co complexation differ in *Glochidion* cf. *sericeum*. We aimed to determine the chemical speciation in frozen hydrated samples of different plant organs, tissues and transport liquid (xylem and phloem) from the root to leaves to gain an understanding of the mechanisms of transport and storage in this species.

## Results

### Herbarium XRF survey and taxonomical status of the study species

*Glochidion* is a very speciose genus of trees and shrubs with 200–300 species occuring from India to South China, Southeast Asia, Malesia, Australia and the West Pacific^35^. In total, 33 species have been enumerated from Borneo^36^. An Herbarium X-ray Fluorescence (XRF) scanning survey was undertaken at the Forest Research Centre (FRC) Herbarium on all holdings of *Glochidion* (totalling 643 specimens). The XRF scanning revealed the existence of several Ni hyperaccumulator species which have been previously reported^11^, and anomalously (>300 μg g^−1^ corrected XRF values) high Co values in two unidentified *Glochidion* specimens with 307 μg g^−1^ and 681 μg g^−1^ Co respectively, and in a specimen of *Glochidion elmeri* which had 539 μg g^−1^ Co (Suppl. Table 1). Herbarium specimens of *G*. cf. *sericeum* (held at the Sabah Parks Herbarium) were also measured with XRF and Co reached up to 951 μg g^−1^ Co in 19 leaves from the three specimens measured. Subsequent bulk analysis with ICP-AES of fragments obtained from these herbarium specimens gave mean values of 1159 ± 217 μg g^−1^ for Co and 2037 ± 205 μg g^−1^ for Ni.

**Figure 1:**
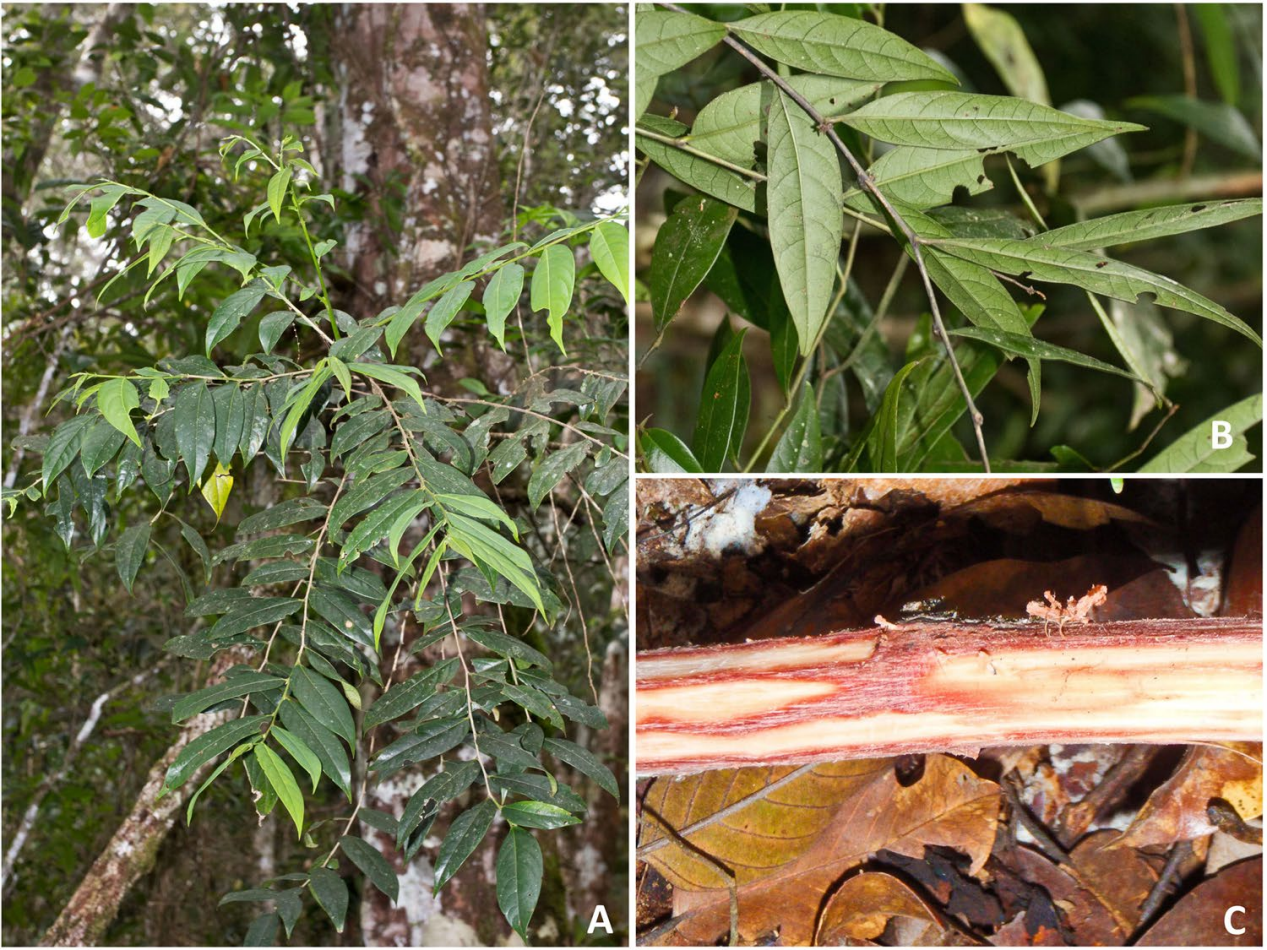
*Glochidion* cf. *sericeum* in the native habitat in Sabah (Malaysia): **(A)** mature understory tree individual near Serinsim; **(B)** detail of leaves of *G*. cf. *sericeum*; **(C)** stripped bark revealing red-coloured phloem tissue rich in Co.

*Glochidion* cf. *sericeum* is a phyllanthoid branching medium-sized tree (5–8 m-high with a stem up to 12 cm in diameter) that grows in the understory of lowland primary mixed Dipterocarp forest (Fig. 1). This taxon is presently known from only one locality (‘Serinsim’) in the Northern Part of Kinabalu Park in Sabah (Malaysia), and we have not been able to find any other occurrences of this taxon to date. This species is rather inconspicuous but locally fairly common (>100 individuals seen) at the site. The leaves are lanceolate (8–15 × 3–7 cm), with acuminate tip and a glabrous appearance. The inner bark (phloem) has a red colour when fresh. The flowers are ~2.5 mm diameter, white-yellowish occurring fascicled in nodes. The fruits are ~4 mm in diameter, pink-red and densely tomentose and open completely septicidal. The taxonomic identity of this *Glochidion* taxon remains elusive and it could not be matched to any of the collections at the FRC Herbarium with 25 identified *Glochidion* species. It appears to be part of the *G. rubrum* Blume group, which is extremely variable, and is possibly allied to *G. azaleon* Airy Shaw from East-Kalimantan (pers. comm. Peter van Welzen). It most likely represents a hitherto undescribed taxon, but insufficient fertile (*i.e.* male flowers en fruits) material has been collected thus far to warrant typification.

### Habitat and soils associated with *Glochidion* cf. *sericeum*

The habitat of *G*. cf. *sericeum* is on deep, strongly laterised soils (Ferralsols) derived from ultramafic bedrock. The Serinsim area (Northern part of Kinabalu Park) is a plateau with soil depth in excess of 10 m and consists primarily of red iron-oxyhydroxides (goethite) with minor Fe-crust (‘ferrocrete’ which consists mainly of hematite). Waterlogging can occur locally due to the formation of this Fe-crust. The soils are acid with pH 3.8–5.7 (mean of pH 4.7) due to the lack of basic ions (Ca, Mg) which have leached and this low pH promotes phytoavailability of Mn and Co^37^. The Fe content of the soils ranges from 19–54 Wt% with 0.8–2.2 Wt% Cr and 1.9–3.9 Wt% Al. Total concentrations of Ca (mean 561 μg g^−1^), K (mean 83 μg g^−1^) and Mg (mean 512 μg g^−1^) are depauperate due to intensive leaching (annual rainfall exceeds 3000 mm). The soil surface is devoid of organic litter and shallow tree roots are abundant; this is indicative of strong biological recycling of these essential macro nutrients by re-uptake in the vegetation. The total concentrations of Co (mean 50 μg g^−1^), Ni (mean 2452 μg g^−1^) and Mn (mean 2318 μg g^−1^) are typical for ultramafic soils. Dilute Sr(NO_3_)_2_-extractable and DTPA-extractable concentrations of Co, Mn, and Ni are rather low compared to the values reported by van der Ent *et al*. (2015) for many soils associated with Ni hyperaccumulator plants in Sabah (for example, the mean DTPA-extractable Ni in the Sabah soils is 117 μg g^−1^). The low extractability and small difference between the Sr(NO_3_)_2_-extractable and DTPA-extractable Ni concentrations (Table 1) are suggestive of occlusion of Ni into well-crystallised Fe-oxides. The Co concentrations vary more widely (mean 0.2 μg g^−1^ vs 1.4 μg g^−1^), and it is possible that, during inundation of the plateau during rain events, a pulse of higher Co is released from Mn-oxides (such as birnessite, (Na_0.3_Ca_0.1_K_0.1_) (Mn^4+^, Mn^3+^)_2_O_4_·1.5 H_2_O).

**Table 1:** Total and extractable elemental concentrations in the soils of the habitat of *Glochidion* cf.

	Al	Ca	Co	Cr	Fe	K	Mg	Mn	Ni	P	Zn
	Wt%	μg g^−1^	μg g^−1^	Wt%	Wt%	μg g^−1^	μg g^−1^	μg g^−1^	μg g^−1^	μg g^−1^	μg g^−1^
*Total*	1.93–3.85	466–723	2–140	0.76–2.18	19.24–53.50	35–114	317–722	882–3425	1413–3698	141–378	152–373
	3.08	561	50	1.63	38.57	83	512	2318	2452	279	270
	**Al**		**Co**	**Cr**	**Fe**	**K**	**Mg**	**Mn**	**Ni**	**P**	**Zn**
	**μg g** ^**−1**^		**μg g** ^**−1**^	**μg g** ^**−1**^	**μg g** ^**−1**^	**μg g** ^**−1**^	**μg g** ^**−1**^	**μg g** ^**−1**^	**μg g** ^**−1**^	**μg g** ^**−1**^	**μg g** ^**−1**^
*Sr(NO*_*3*_ *)*_*2*_	0.5–31		0.04–0.7	0.1–0.6	0.2–4.1	8.5–58	5.0–1190	4.0–902	0.1–15	0.3–30	1.3–17
	8.6		0.2	0.3	1.8	36.6	115	87	3.2	10	5.6
*DTPA*	1.2–83		0.4–4.0	0.05–1.3	10–164	7.7–50	2.9–23	1.2–57	0.4–8.1	0.2–1.0	0.1–0.4
	27		1.4	0.6	64	33	15	16	3.7	0.6	0.3

*sericeum* in Sabah (Malaysia), the values are ranges (above) and means (below) in μg g^−1^ and Wt% as indicated (*n* = 15 soil samples). The digests and extracts were analysed with ICP-AES.

### Bulk elemental concentrations in plant tissues of *Glochidion* cf. *sericeum*

Samples were collected from various parts of *G*. cf. *sericeum* trees in the habitat at Serinsim, including roots, wood, twigs, leaves, and fruits. Phloem tissue was also extracted from the main trunk. The leaves have relatively high concentrations of Ca (mean 2725 μg g^−1^), K (mean 3453 μg g^−1^) and Mg (mean 6323 μg g^−1^) (Table 2) compared to the concentrations of these elements in the local soil (Table 1). In the fruits, Ca concentrations (658 μg g^−1^) are lower, whereas K (5949 μg g^−1^) and P (941 μg g^−1^) are higher in comparison to the other tissues. The relative enrichment of K, P and S in the twigs compared to the wood may be attributed to the presence of phloem tissue (which is enriched in these elements) in the twigs. Compared to many other Ni hyperaccumulator plants from Sabah^34^, the leaves of *G. sericeum* do not contain high Ni concentrations (mean is 1602 μg g^−1^), making it a weak Ni hyperaccumulator. However, the foliar concentrations of Co are unusually high at 159–1311 μg g^−1^ (mean is 482 μg g^−1^). As such the mean foliar Ni:Co quotient is 3.3 which contrasts with many other hyperaccumulator plants (*e.g.* with the mean Ni:Co quotient of 475 in 24 different Ni hyperaccumulator species from Sabah noted earlier). The phloem tissue is even more concentrated, and can contain up to 2593 μg g^−1^ Co (~0.3 Wt%). The wood, twigs and fruits are Co depleted with 88, 217 and 72 μg g^−1^ respectively. The coarse roots are also comparatively low in Co (mean 73 μg g^−1^), suggesting that Co is effectively translocated to the phloem and the shoot (and ultimately the leaves). The foliar concentrations of Cr, Fe and Zn are unremarkable, but the concentration of Mn is high (mean 2866 μg g^−1^). In the phloem tissue, the concentration of Mn even reaches up to 16 514 μg g^−1^ or 1.7 Wt%. In the roots, Mn, Ni and Zn concentrations are lower than in the leaves, pointing to effective translocation to the shoots. The Fe concentrations are relatively high in the root samples, but this might (partly) be caused by minor contamination of the root surface with soil particles rich in Fe.

**Table 2:** Bulk elemental concentrations in plant tissues (leaves, twigs, phloem, wood, fruits) of *Glochidion* cf.

Sample type	*n*	Al	Ca	K	Mg	P	S
Fruit	1	27	658	5949	3123	941	960
Twigs	1	48	665	7594	2102	197	679
Wood	1	20	405	1405	2527	82	291
Leaves	19	65–482	1593–5365	112–8447	1869–8560	224–442	785–2237
		197	2725	3453	6323	346	1453
Phloem	2	83–125	2928–3900	4194–5422	5355–5405	172–240	927–936
		104	3414	4808	5380	206	931
Roots	2	100–264	1409–4951	1432–1630	4759–5160	80–97	1585–2324
		182	3180	1531	4959	89	1954
**Sample type**	***n***	**Co**	**Cr**	**Fe**	**Mn**	**Ni**	**Zn**
Fruit	1	73	16	42	169	174	23
Twigs	1	217	8	20	1104	282	40
Wood	1	88	15	11	295	235	16
Leaves	19	159–1311	0.1–83	21–63	257–2999	831–2447	9.6–103
		482	43	38	2866	1602	36
Phloem	2	115–2593	3.9–23	27–40	1686–16514	473–1646	52–171
		1354	14	34	9100	1059	112
Roots	2	30–116	34–75	773–2654	805–856	149–393	52–93
		73	54	1713	830	271	73

*sericeum* from Sabah (Malaysia), the values are ranges (above) and means (below) in μg g^−1^ dry weight analysed with ICP-AES.

### Elemental distribution in mature leaves of *G*. cf. *sericeum*

**Figure 2:**
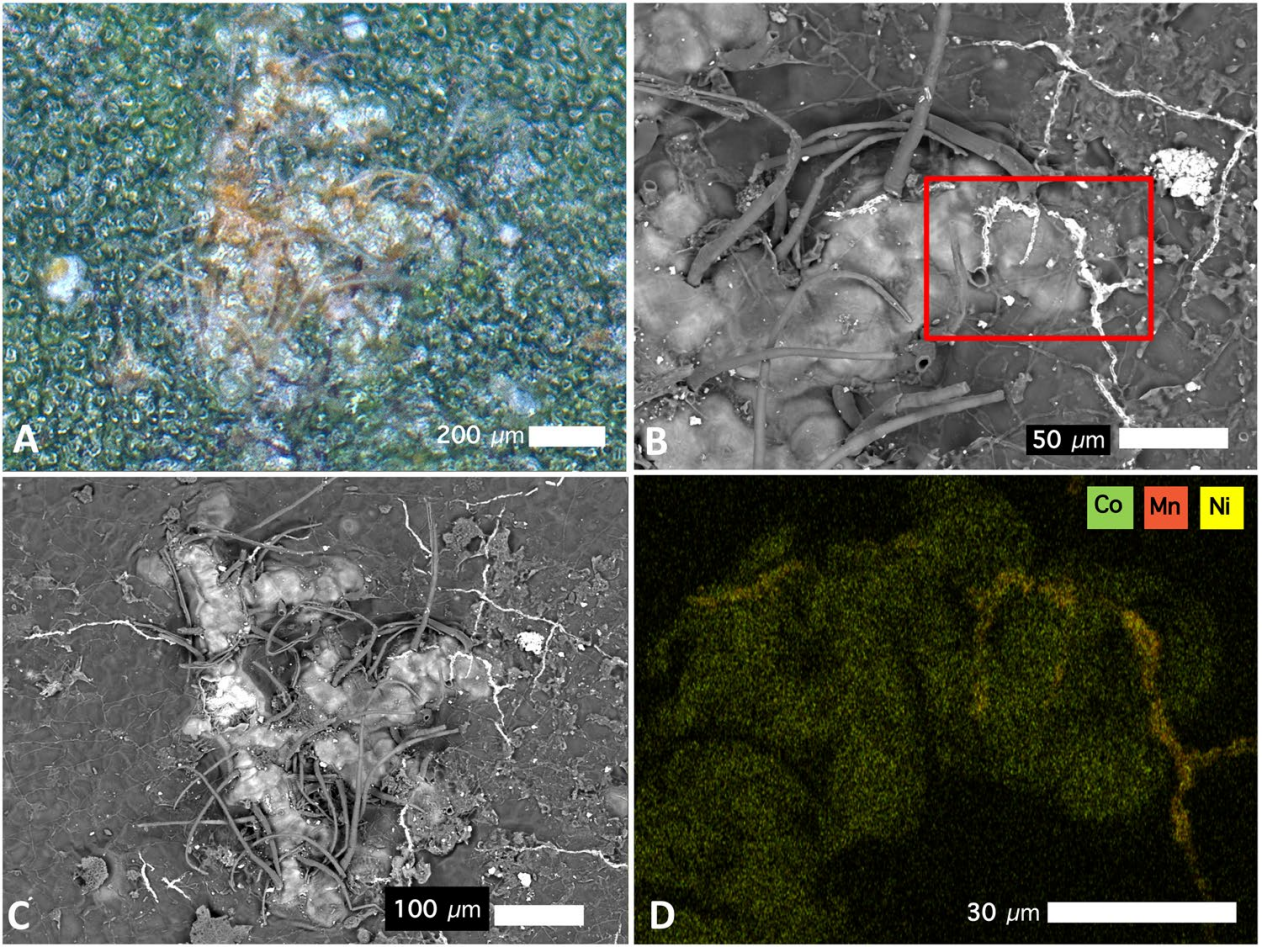
Cobalt-rich surface lesions on the abaxial leaf surface of *Glochidion* cf. *sericeum*: **(A)** bright-field light microscopy image showing brown lesions developed under the cuticle; **(B)** Scanning Electron Microscopy (SEM) image showing the same lesion with trichomes; **(C)** SEM image of the same lesion, full view; **(D)** Energy-Dispersive Spectroscopy (EDS) image of the lesion with rectangular box outline field-of-view in relation to the SEM image in b. Key to colours: Green is ***Co***, orange is ***Mn***, yellow is ***Ni***. Scale bars denote 30, 50, 100 and 200 μm.

**Figure 3:**
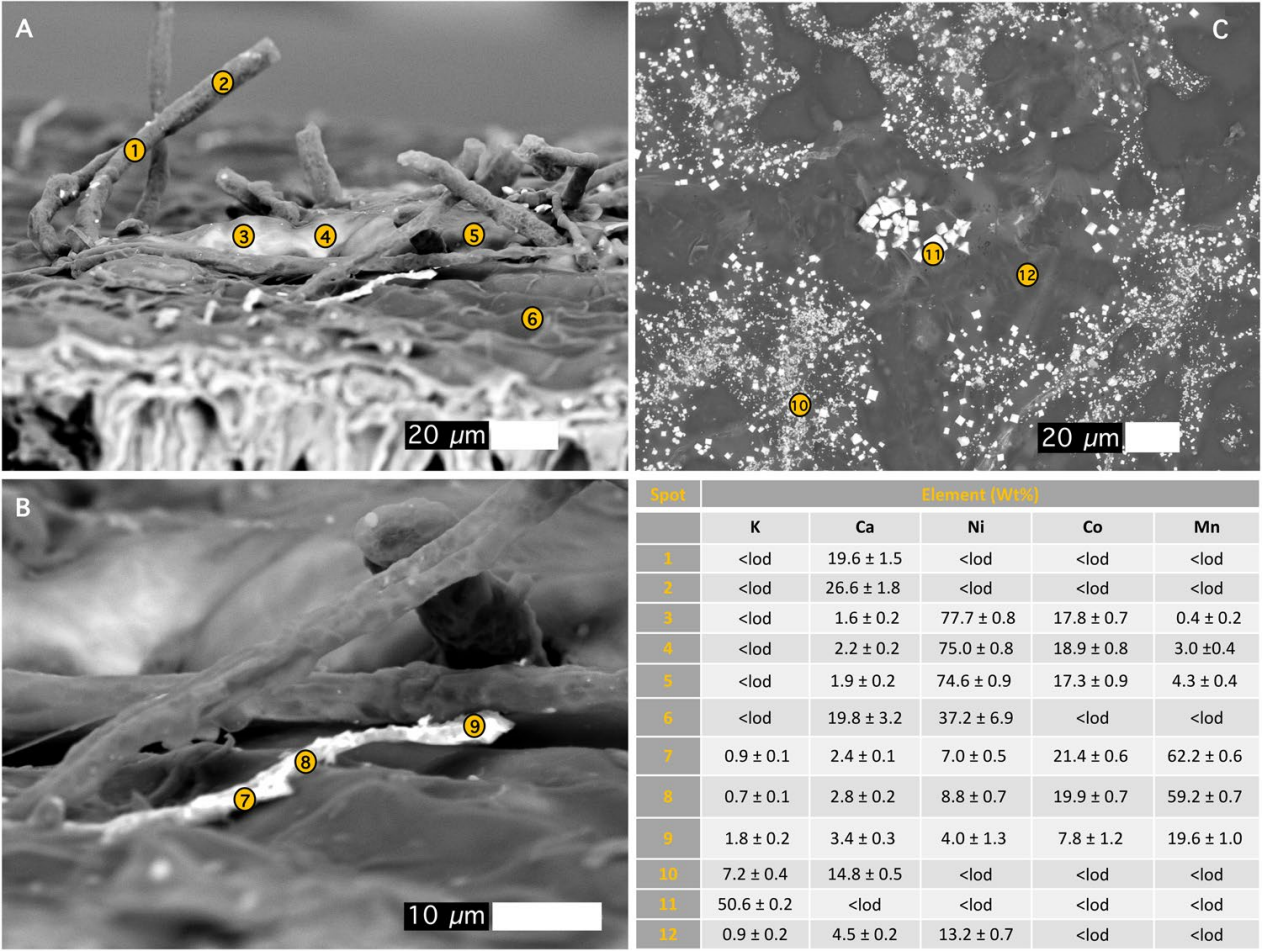
Scanning Electron Microscopy (SEM) images of leaf surface with trichomes. Energy-Dispersive Spectroscopy (EDS) point analyses, with concentrations in Wt% and errors of analysis (±σ uncertainty) and < *lod* demotes below instrumental limit of detection. Scale bars denote 10 and 20 μm.

The abaxial side of *G*. cf. *sericeum* mature leaves have small brown spots that are visible to the naked eye, and which appear as ‘blister’-like lesions (filled with a brown solid) with trichomes evident under a bright field microscope (Fig. 2A). Magnified further under a Scanning Electron Microscope (SEM) the lesions appear to have formed directly under the cuticle by the exudation of a substance with a high density (refer to the Backscatter Electron Image, BSE). Trichomes protrude through the lesions with a high-density precipitate channelling over the surface of the cuticle (Fig. 2B,C). Energy-dispersive spectroscopy (EDS) analysis shows that the substance in the lesion is strongly enriched in Ni and Co, whereas the ‘veins’ are very high in Mn (Fig. 2D). EDS point analysis was undertaken on the same mature leaf to identify the major elemental composition of the lesion deposits and the ‘veins’ on the leaf surface. Figure 3A shows an oblique view of the leaf surface close to a fractured lesion. It clearly shows the protruding trichomes and the blister-like lesions, as well as the ‘veins’. Point analysis reveals that tubular trichomes are composed mainly of Ca, whereas the lesion deposit is extremely high in Ni and also Co, but relatively low in Mn. Figure 3B shows a close-up of these ‘veins’ which measure roughly 2 μm in diameter and are about 50 μm long. The image is a backscatter electron (BSE) scan, in which the electron beam reacts elastically with atoms in the sample, and because high atomic number *Z* are more strongly scattered than lighter atoms, higher-*Z* elements show up with higher contrast (*i.e*. brighter tones). The ‘vein’ is clearly a mineral deposit made up almost entirely of high-*Z* elements, with the EDS showing that it is mainly composed of Mn, with minor Co and Ni. Figure 3C shows an area of the foliar surface with crystalline mineral deposits likely originating from guttation fluids exuded by hydathodes. The composition is mainly KCl (chloride values not shown but 45.5 ± 0.1 Wt% in measurement point 11).

**Figure 4:**
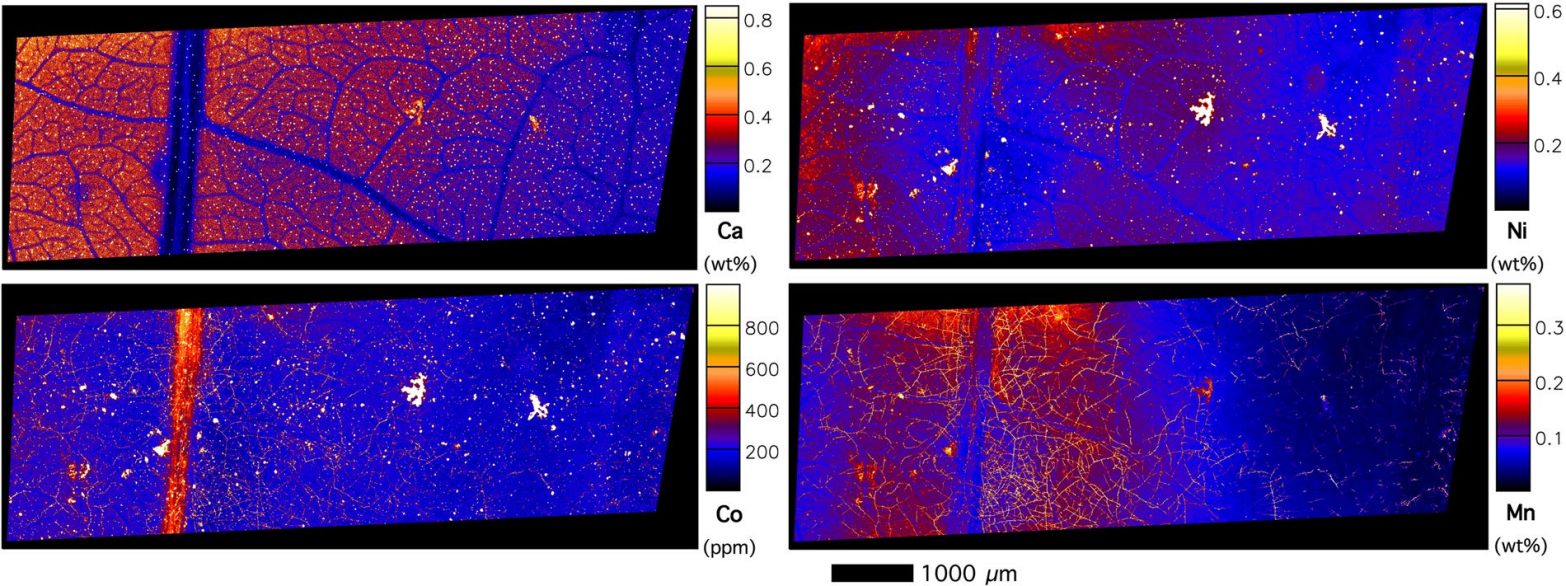
Micro X-Ray Fluorescence (μXRF) image of a *Glochidion* cf. *sericeum* leaf displaying elemental maps for Ca, Mn, Co and Ni. Scan area is 14.9 × 9 mm, with a step size of 2 μm and a per-pixel dwell of 0.98 ms. The maps were flattened to the Compton map to correct for differences in sample thickness and density. Scale bar 1000 μm.

**Figure 5:**
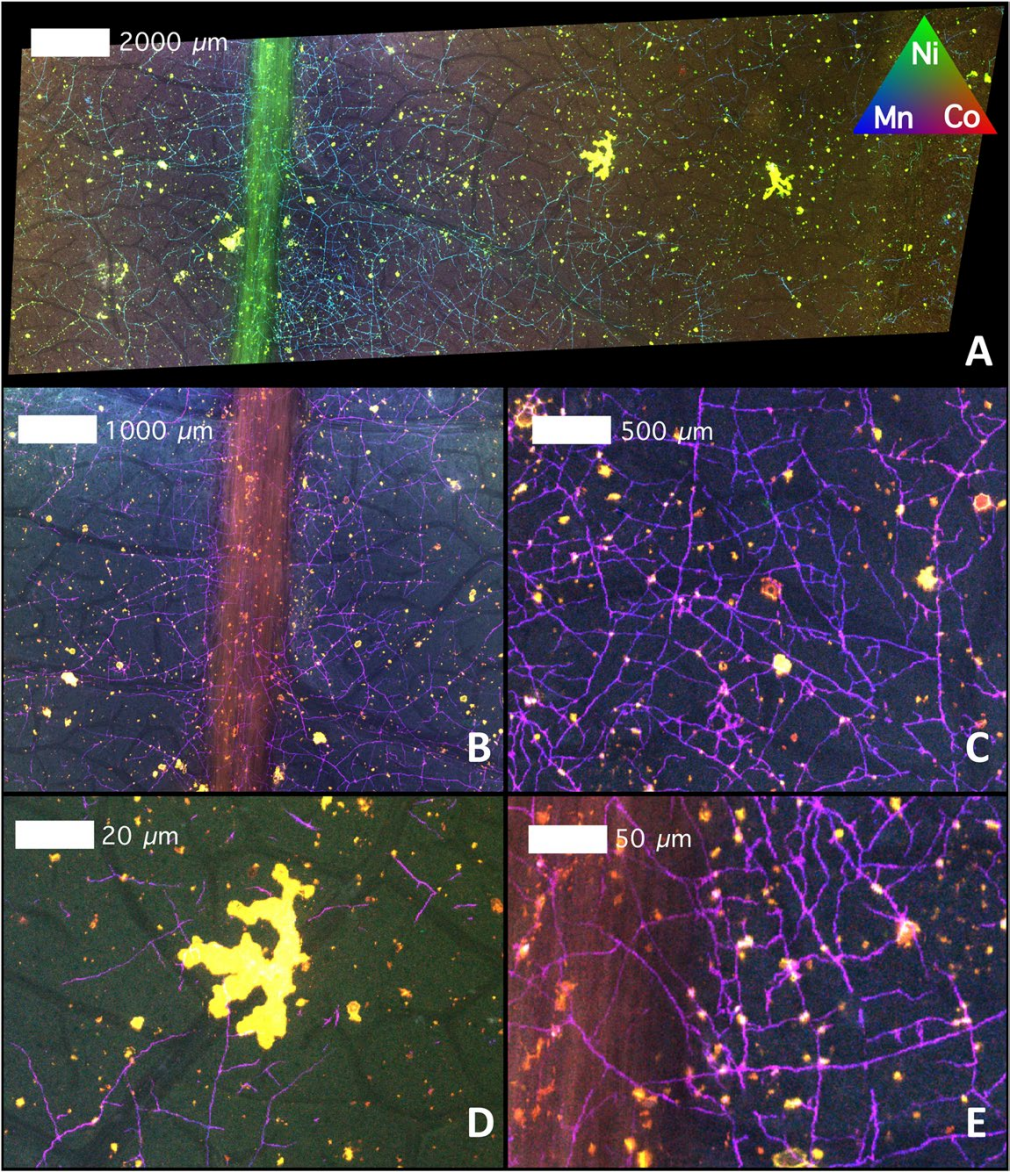
Tri-colour μXRF image (Key to colours: red is ***Co***, green is ***Ni***, blue is ***Mn***) of the same *Glochidion* cf. *sericeum* leaf from Fig. 3. Panels B–E shows details. Scale bars denote 20, 50, 500 and 1000 and 2000 μm.

**Figure 6:**
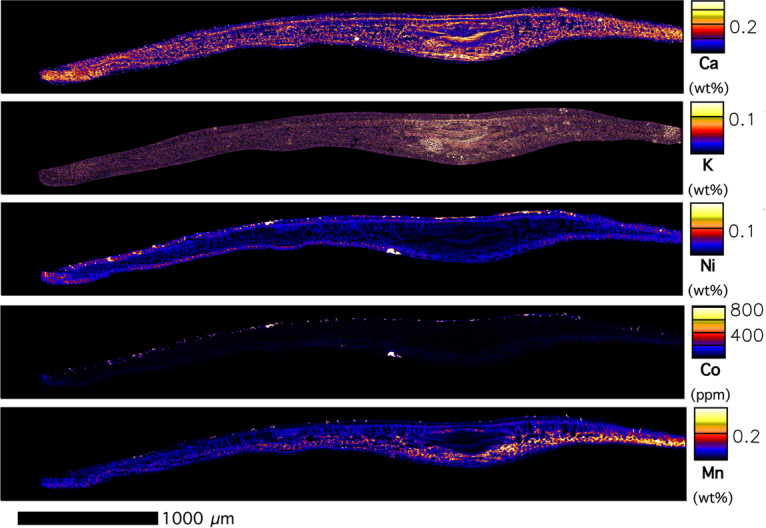
μXRF image of a frozen-hydrated *Glochidion* cf*. sericeum* leaf cross-section displaying elemental maps for Ca, K, Ni, Co and Mn. Area: 4.90 × 0.66 mm, with a step size of 1 μm and a per-pixel dwell of 1.30 ms. The maps were flattened to the Compton map to correct for differences in sample thickness and density. Scale bar 1000 μm.

**Figure 7:**
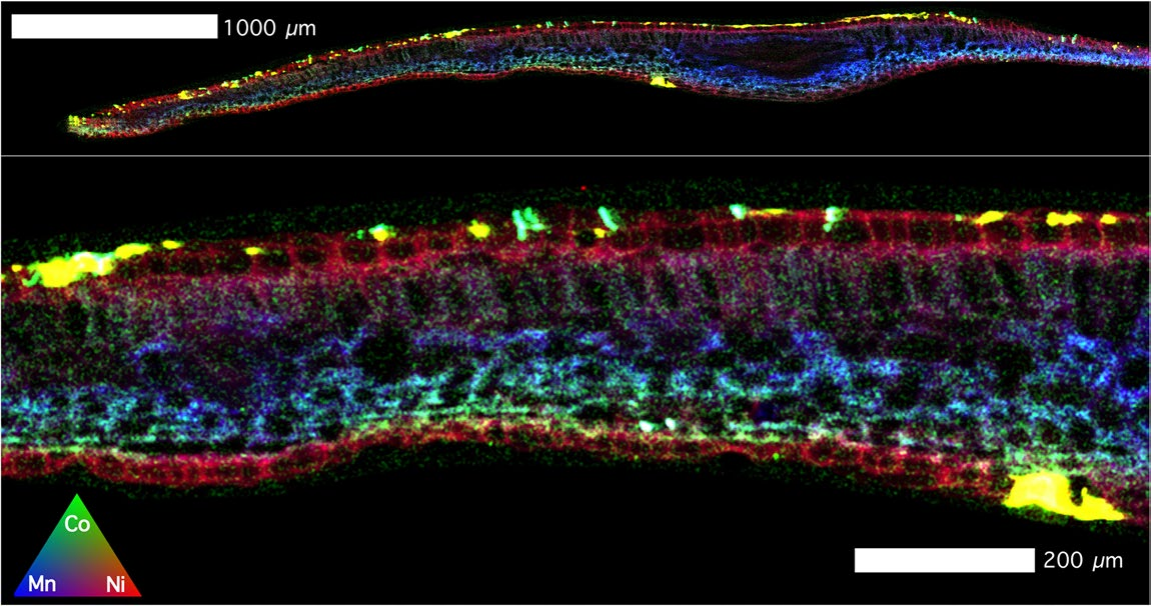
Tri-colour μXRF image (Red is ***Ni***, Green is ***Co***, Blue is ***Mn***) of the same *Glochidion* cf. *sericeum* leaf section from Fig. 5. Lower panel shows detail. Scale bars denote 200 and 1000 μm.

**Figure 8:**
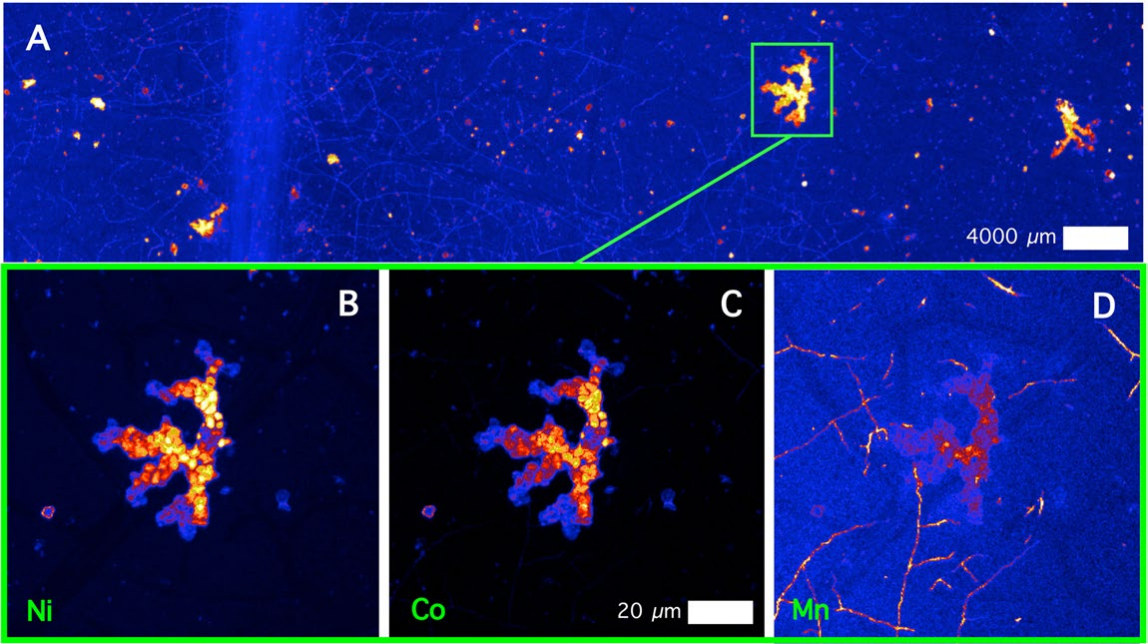
Details of μXRF images of a Ni-Co rich lesion. The top panel shows a Ni elemental map, whereas the lower panels show elemental maps (Ni, Co, Mn), with de-saturated contrast to show the ‘nodular’ structure. Scale bars denote 20 and 4000 μm.

The lesions feature dominately in the XFM images (Figs 4–8) with extreme enrichment in Ni and Co. Note that the electron microscope images (Figs 2 and 3) were obtained from the exact same sample that was previously imaged with XFM (compare with Figs 4 and 5). In cross-sections (Figs 6 and 7 and Suppl. Figure 6), adaxial epidermal cells and mesophyll cells are clearly visible and it is clear that Ni is concentrated in the apoplastic space surrounding the adaxial and abaxial epidermal cells of the leaf. In contrast, Co is concentrated mainly in hotspots under the epidermal cells, and locally spreading through the epidermal cells reaching the cuticle, thereby forming the lesions observed in the bright field and electron microscopy. The distribution of Mn is very different again, with most Mn concentrated in the spongy mesophyll, as well as in trichomes at the adaxial side of the leaf. Potassium is enriched in phloem bundles of the central vein of the leaf, whereas Ca is depleted in all veins. Zinc is also depleted in the vasculature of the leaf. Chromium is located mainly in the central vein, and in the prominent lesion, although the concentrations are relatively low [compared to Ni and Co].

The panels in Fig. 8 show detailed XFM elemental maps of one of the largest lesions on the leaf surface. The composition of all 4 lesions is rather similar, and composed mainly of Ca (likely the trichome) and Mn, Co and Ni (the lesion deposit). Lowering the contrast in the elemental maps of Ni, Co and Mn of the lesion reveals a globular structure packed with nodules (refer to Ni, Mn and Co maps). This may point to repeated exudation of small amount of liquid over time. In contrast, even though there is some Mn in this lesion, the surficial ‘veins’ are clearly visible too, which have extreme Mn enrichment. The highest Ni and Co concentrations co-occur in the main lesions across the foliar surface.

### Chemical speciation of Ni and Co in *Glochidion* cf. *sericeum*

**Figure 9:**
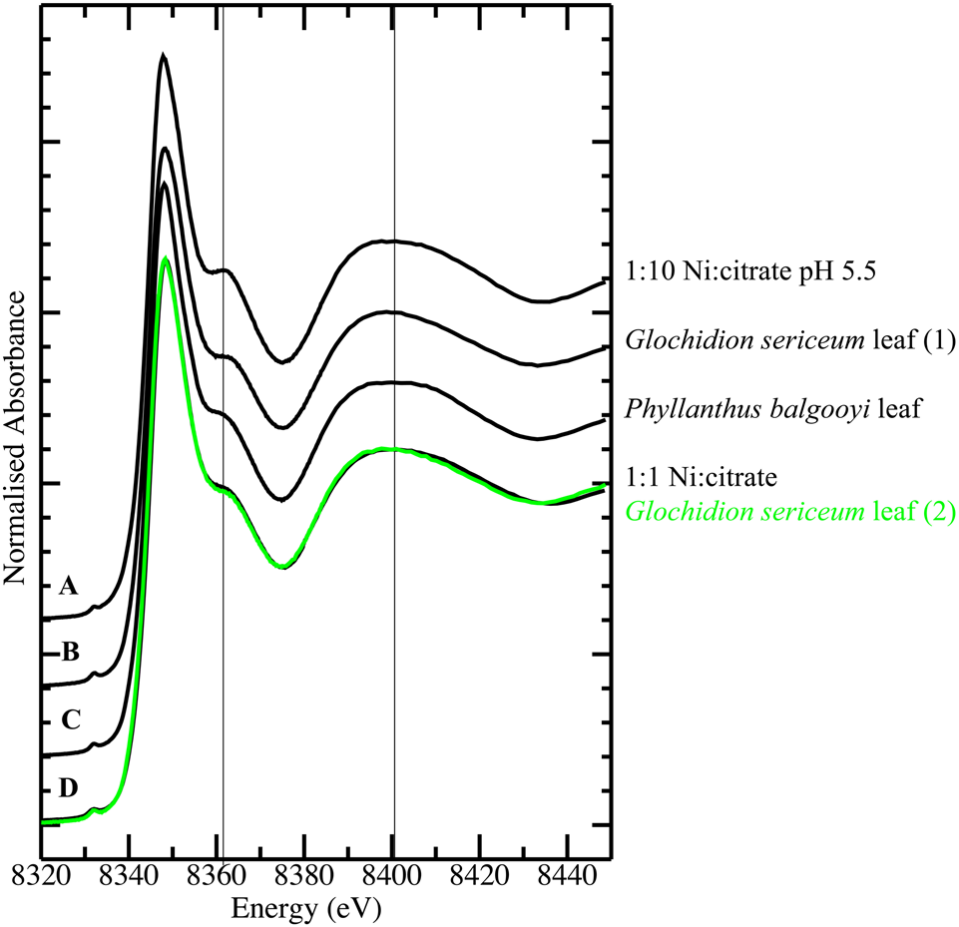
Ni K-edge X-ray absorption near edge spectra (XANES) for (A) 1:10 Ni:citrate in buffered pH 5.5 aqueous solution; (B) *Glochidion* cf. *sericeum* leaf sample 1; (C) *Phyllanthus balgooyi* leaf tissue (from^33^); (D) *Glochidion* cf. *sericeum* leaf tissue sample 2 (green trace), 1:1 Ni:citrate in buffered pH 5.5 aqueous solution (black trace). Vertical lines are drawn at 8361 and 8400 eV to aid visual comparison of key features in the spectra.

Bulk chemical speciation of Ni in leaves and Co in leaves, phloem and roots were determined with X-ray absorption spectroscopy (XAS), by comparing the XANES spectra against the range of solution standards described above. The Ni K-edge spectra from two leaf samples from *G*. cf. *sericeum* were found to be identical to each other and to be very similar to those recorded from the tissues of *P. balgooyi*^33^, and to the spectrum of a 1:1 mixture of Ni and citrate in pH 5.5 buffered aqueous solution (Fig. 9). The Ni spectra of the *G*. cf. *sericeum* leaves differed marginally from that of the *P. balgooyi* leaves in that the broad peak centred at ~8400 eV in the *G*. cf. *sericeum* spectrum was slightly less broad and less symmetric than that in the spectrum of the *P. balgooyi* leaves. Attempts at fitting a linear combination of model compound spectra to these tissue spectra were unsuccessful for the same reasons as previously noted^33^ – specifically, a principal component analysis was not conclusive regarding the number of model compounds that should be included in the linear regression and fit results generated with different numbers of components varied markedly. However the fit attempts suggested a contribution from the Ni:tartrate model of approximately one third and this would explain the noted difference in the Ni spectra between *P. balgooyi* (all 1:1 Ni:citrate) and *G*. cf. *sericeum* leaves (a mixture of 1:1 and 1:10 Ni:citrate and either the Ni:tartrate or Ni:malate with the spectra of the last two being indistinguishable).

**Figure 10:**
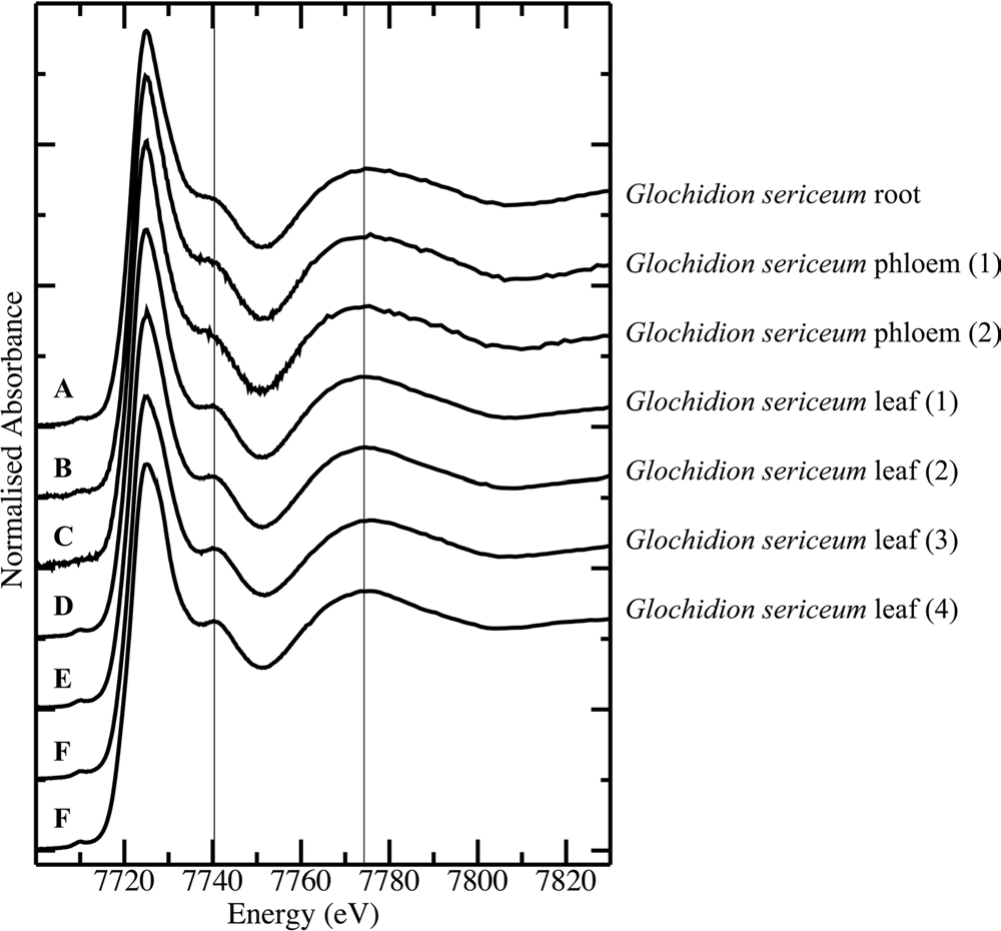
Co K-edge X-ray absorption near edge spectra (XANES for (A) *Glochidion* cf*. sericeum* root tissue; (B,C) *Glochidion* cf. *sericeum* phloem samples from separate individuals; (D–G) *Glochidion* cf*. sericeum* leaf samples from separate individuals.

**Figure 11:**
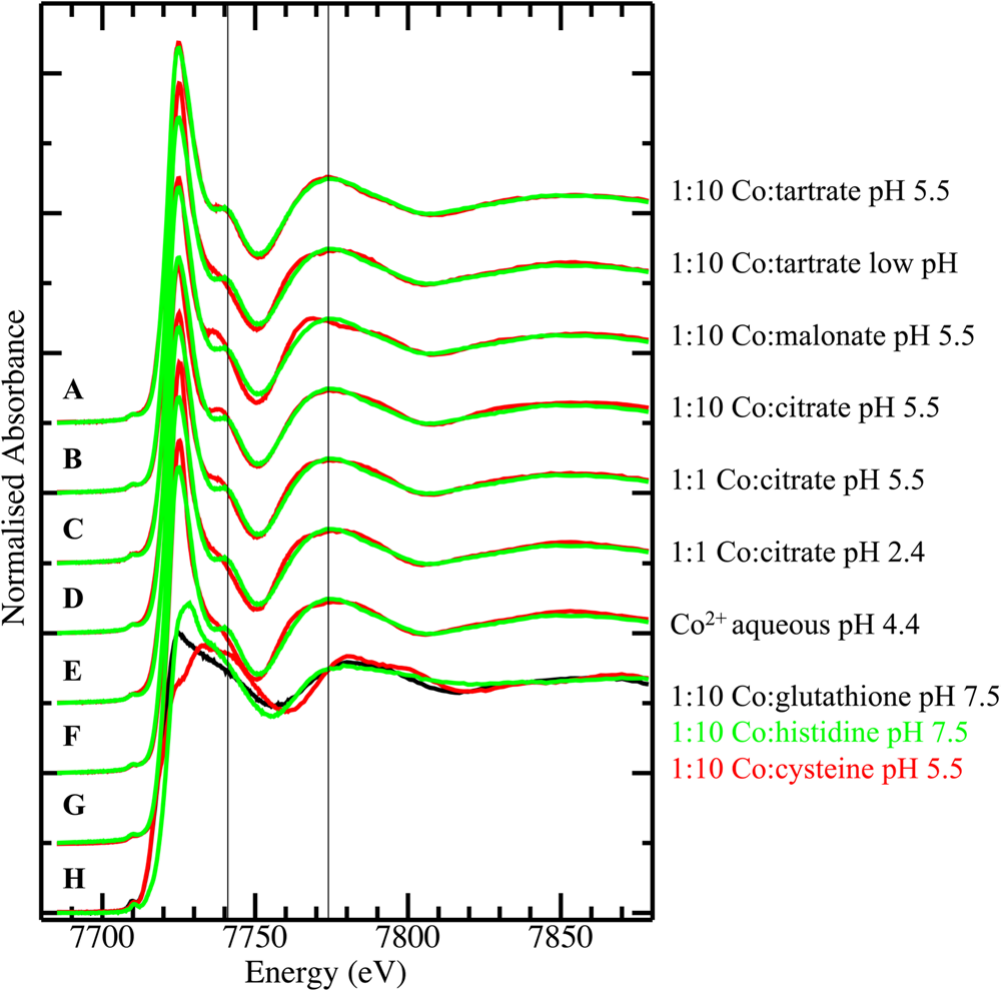
Co K-edge X-ray absorption near edge spectra (XANES) for (A–H) *Glochidion* cf. *sericeum* leaf tissue 17 (green trace drawn repeatedly) compared against (red traces) 5 mM Co(II) model spectra; (A) 1:10 Co:tartrate in buffered pH 5.5 aqueous solution; (B) 1:10 Co:tartrate in unbuffered aqueous solution; (C) 1:10 Co:malonate in buffered pH 5.5 aqueous solution; (D) 1:10 Co:citrate in buffered pH 5.5 aqueous solution; (E) 1:1 Co:citrate in buffered pH 5.5 aqueous solution; (F) 1:1 Co:citrate in unbuffered pH 2.4 aqueous solution; (G) aqueous Co(II) at pH 4.4; (H) 1:10 Co:glutathione in buffered pH 7.5 aqueous solution (black trace), 1:10 Co:histidine in buffered pH 7.5 aqueous solution (green trace), 1:10 Co:cysteine in buffered pH 5.5 aqueous solution (red trace).

Co K-edge spectra across *G*. cf. *sericeum* leaf samples from 4 separate individuals, one root and two phloem samples from separate individuals, were difficult to distinguish by visual inspection (Fig. 10), although the signal-to-noise level in the phloem spectra reflected the significantly lower Co concentration in the fluid compared to other tissues. Comparison of the Co K-edge spectrum of a representative of the plant tissue set (*G. sericeum* 1) against the spectra of a series of model compound spectra revealed an essentially identical match to 1:10 Co:tartrate at pH 5.5 and considerably poorer matches to all other models. The set of model compound spectra was reduced to those shown in Fig. 11 by identifying subsets of indistinguishable spectra. The spectra of the pH unadjusted 1:10 Co:malate, 1:10 Co:nicotiamide, 1:10 Co:malonate, 1:10 Co-succinate and the pH 5.5 1:10 Co:glutamate models were all identical to that of the pH unadjusted 1:10 Co:tartrate spectrum, while the spectra of the pH 5.5 1:10 Co:tartrate and malate solutions were distinct from these, but identical to each other. The spectrum of the pH unadjusted tartrate complex was chosen to represent the first of these sets of compounds with identical spectra in the XANES fitting, while the pH 5.5 tartrate model spectrum was used to represent itself and the identical pH 5.5 malate complex. This choice was made due to the tartrate spectra having the best signal-to-noise. The net result is that all complexes noted in the Methods section were eliminated as significant components by the XANES fitting procedure, except the pH 5.5 tartrate and malate complexes which cannot be distinguished by this method. We note that inclusion of compounds with identical spectra in the XANES fitting procedure will lead to spurious results. There are some minor variations amongst the Co spectra of the plants - the peak at 7740 eV varies a little in intensity and this may potentially be explained by a variation in relative compositions of 1:1 and 1:2 tartrate complexes.

Least-squares linear combination fits of the set of seven closely-matched model compound spectra from Fig. 11 to the plant tissue Co spectra shown in Fig. 10 resulted in identification of 1:10 Co:tartrate as the only component in every case – all other potential components were eliminated by the fitting.

Close inspection of the “white line” peak of the Co spectra of the plant samples showed a broadening of the peak in some cases with a shoulder occasionally evident on the high-energy side of the peak (arrow in Suppl. Figure 4). The energy of the shoulder at about 7727 eV is consistent with O-bound Co^3+^ species^38^ but given that the fractional contribution of this species to the total Co speciation pool was inconsistent and very low, Co^3+^ model spectra were not included in the fitting procedure. A principal component analysis of the seven plant tissue spectra indicated that all spectra were extremely similar, as evident by eye, but that one or two minor components were present in addition to the dominant one. Inspection of the principal component eigenvectors indicated that the presence of Co^3+^ in some of the tissue samples was responsible for this variation with the intensity of the second and third eigenvectors focussed on the “white line” peak (Suppl. Figure 5).

## Discussion

The bulk chemical speciation of Ni and Co is rather similar in all the tissues and transport liquids, and associated with carboxylic acids. The Ni K edge XANES results from *G*. cf. *sericeum* are similar to those reported from *Phyllanthus balgooyi, P. securinegioides* and *Rinorea balgooyi*^33^ and match those of a 1:1 mixture of Ni and citrate in pH 5.5 buffered aqueous solution (but with the possible addition of a minor component of a tartrate complex). The XANES spectra of Ni ligated with carboxylic acids have similar features to that of the hydrated Ni(H_2_O)_6_^2+^. That shows that in these complexes, Ni is octahedrally coordinated with an inner coordination geometry that is not significantly altered upon complexation with carboxylic ligands^32,39^. The chemical speciation of Co is associated with either coordination by tartrate or malate (XAS does not distinguish between these two species on the basis of their spectra). Whereas tartrate is typically present at low concentrations in plant leaves, malate is generally present at higher concentrations as the intermediate of the tricarboxylic acid (Krebs) cycle and has a multitude of functions in plant metabolism. It therefore appears more likely that Co is complexed with malate rather than tartrate in *G*. cf. *sericeum*.

Apart from the intensively studied Ni-Cd-Zn hyperaccumulator *Noccaea caerulescens*^40,41^, simultaneous hyperaccumulation (involving Ni and/or Co) is an understudied phenomenon. In the Ni hyperaccumulator *Alyssum murale* Ni accumulates in the epidermal tissue of the leaves, whereas Co is excreted from the apoplast and forms Co-rich mineral precipitates on the foliar surface^42^. A similar extracellular detoxification mechanism for Co was noted in oat plants^26^. These observations align with the results of this study on *G*. cf. *sericeum* that shows it has epidermal Ni accumulation and Co exudation on the leaf surface in the form of lesions. The oxidation to Co^3+^ on the surface of leaves after exudation appears likely and a reasonable explanation for both the presence and variation in the intensity of the Co K edge shoulder in the XAS analysis where exposure to aerial oxygen leads to oxidation from Co^2+^ to Co^3+^. The toxicity response to excess Co somewhat resembles that of Mn, with the formation of high Mn lesions (and eventual necrosis) on leaves^43^. In *Alyssum* spp. Mn is accumulated at trichome bases^44^, whereas in the Mn hyperaccumulators *Gossia bidwillii, Virotia neurophylla, Macadamia integrifolia* and *M. tetraphylla* from Australia and New Caledonia Mn is concentrated mainly in the palisade mesophyll^45,46^. The Mn hyperaccumulator *Gossia fragrantissima* (Proteaceae) simultaneously accumulates Ni and hyperaccumulates Co and Zn, with Zn primarily localized in foliar epidermal cells while Mn and Ni are also concentrated in the palisade layer^47^.

The contrasting localisation of Ni and Co in *G*. cf. *sericeum* is suggestive of different modes of sequestration for these elements. *Glochidion* cf. *sericeum* has evolved Ni hyperaccumulation that consists of effective storage of foliar Ni in the epidermal region, but these adaptations have apparently not conferred a tolerance to endogenous Co. As such, *G*. cf. *sericeum* is a (relatively weak) Ni hyperaccumulator, and copes with excess of Co by exclusion via exudation in the leaves. This response is also found in *Alyssum* spp. which are less tolerant to Co than to Ni, altough Co accumulation occurs at lower soil concentrations^48^. Hence in *Alyssum* spp. mechanisms to cope with potentially phytotoxic Co rely on excretion of Co as insoluble residues on the leaf surface around trichomes, similar to *G. sericeum*. This mechanism may be linked to Mn toxicity responses with the formation of lesions, as in *Alyssum murale*, Ni and Co enrichment occur around trichomes, associated with a Mn-rich zone surrounding the base of the trichomes^49^. The transport and delivery of Ni to the leaves occur as mass flow through the xylem, driven by transpiration, and it may be possible that excess Ni escapes the leaves via leaf venation terminals (hydathodes) in guttation fluids^50^. In the Ni hyperaccumulator *Thlaspi* (*Noccaea*) *japonicum*, data suggest that Ni is translocated along with the transpiration stream and concentrated around stomata, and that the excess amount of Ni is excreted via the guttation fluid^51^.

The strong affinity of Mn-oxides for Co may explain the lower Co mobility in Mn-rich soils^52^. When soils are waterlogged, Co is associated mainly with amorphous Fe-oxides. The ratio of soluble Ni:Co then becomes higher (*i.e*. 5:1) than in typical ultramafic soils (10:1–50:1) and *Berkheya coddii* (Asteraceae) may under these circumstances accumulate >600 μg g^−1^ foliar Co^21^. Cobalt accumulates mostly when plants grow in acidic soils, whereas Ni accumulates when plants grow in neutral soils^53,54^. The uptake of Co in *G*. cf. *sericeum* is likely the consequence of local soil conditions that result in high Co availability considering that the mean soil Ni:Co quotients in the DTPA extract (mean of 2.6) is similar to that in the plant leaves (mean 3.3). We hypothesize that Co uptake occurs due to semi-selective membrane-transport proteins in roots that are part of the Ni hyperaccumulation pathway in this species, and it would be interesting to investigate this further using molecular biology approaches. This response of *G*. cf. *sericeum* differs from many other hyperaccumulators; for example, *Psychotria sarmentosa* has mean (n = 8) Ni and Co concentrations of 12 825 μg g^−1^ and 3.6 μg g^−1^ respectively, translating to a Ni:Co quotient of >3500 (unpublished data). Although the foliar Mn concentrations are locally high in the tissues of *G*. cf. *sericeum*, the accepted definition of hyperaccumulation (*viz*. van der Ent *et al*. 2013) is based on total (bulk) concentration of trace element in the leaves (Mn >10 000 μg g^−1^) and on that basis, *G*. cf. *sericeum* is not a Mn hyperaccumulator.

The feasibility of phytomining has been demonstrated at field scale^16,55–57^, but is limited principally to Ni. Cobalt phytomining was first proposed in the early 1990s as a possibility using Ni-hyperaccumulator species on ultramafic soils^19^, but it was noted that the presence of Ni limits the uptake of Co in most Ni hyperaccumulator plants^58^. *Alyssum* species can grow with shoot concentrations >1000 μg g^−1^ in Co-contaminated soils^58^ and *Berkheya coddii* (Asteraceae) can have foliar Co concentrations of >2000 μg g^−1 ^^59^. *Glochidion* cf*. sericeum* is unlikely to be a good candidate for Co phytomining on account of its habitat requirements (understory rainforest niche), presumed slow-growth rate, and relatively low Co concentrations in its overall biomass (especially the woody parts). However, this species does provide an interesting model to study the phenomenon of ‘simultaneous hyperaccumulation’ and this research shows that tolerance to one metal (Ni) does not confer tolerance to another (Co), and that different tolerance mechanisms may operate to avoid phytotoxicity.

## Materials and Methods

### Herbarium XRF scanning

The Niton XL3t 980 analyser (Thermo-Fisher Scientific) uses a miniaturised X-ray tube (Ag anode; 6–50 kV, 0–200 µA max) as its main excitation source. The X-ray tubes irradiates the sample with a stable source of high-energy X-rays, and fluorescent X-rays are detected, identified and quantified by a Silicon Drift Detector (SDD). In order to calibrate the XRF instrument, a total of 590 dried leaf samples (6 mm ∅ leaf discs) were analysed with XRF. The dried leaf samples originated from an ecological study undertaken in Sabah in 2010–2014^60^. After XRF measurement, the leaf discs were weighed and digested and with ICP-OES as described below for the plant tissue samples. In total, 169 samples exceeded the instrumental limit of detection (for XRF) for Co and 533 samples for Ni and these values were used for the regression analysis described below. A lower limit cut-off was set at 300 μg g^−1^ for Ni XRF raw values and at 175 μg g^−1^ for Co XRF raw values >300 μg g^−1^ within the linear fit of the regression (Ni R² 0.73013, Co R² 0.78684). The XRF and ICP-AES values were then regressed (*Ni corrected* = *(1.07*36*XRF) + *10*2*2.1 Ni* and *Co corrected* = (0.3371*XRF) + *165.71*) and the formulas applied to the XRF raw data to obtain ‘corrected values’.

In total, 643 *Glochidion* specimens (covering 25 different species) were measured at the herbarium of the Forest Research Centre (FRC) in Sepilok, Sabah, Malaysia. Each specimen was measured for 30 seconds in ‘Soil Mode’ and the regression equations were applied to the raw XRF data to obtain equivalent ICP-AES estimates.

### Collection of plant tissues and soils

The tissues samples of *G*. cf. *sericeum* were collected in the north of Kinabalu Park (near Serinsim) from the natural habitat in Sabah (Malaysia). The tissue samples intended for synchrotron XAS analysis were excised with a razor blade, placed in polycarbonate cuvettes, covered with Kapton tape and rapidly frozen by immersion in liquid N_2_ (−196 °C) in the field. Tissue samples intended for XFM analysis were similarly excised, but immediately shock-frozen using a metal mirror technique in which the samples were pressed between a block of copper (Cu)-metal cooled by liquid N_2_ and a second cooled Cu-metal block attached to a Teflon holder. This ensured extremely fast freezing of the plant tissue samples to prevent cellular damage by ice crystal formation. The samples were then wrapped in aluminium (Al) foil, and both XAS and XFM samples were transported and subsequently stored in a cryogenic container (at least −190 °C) until further processing (*e.g*. sectioning and mounting for XFM analysis as described below).

The tissue samples for XFM analysis were cryo-transferred on dry ice (−80 °C) into the cryo-chamber of the microtome (maintained at −30 °C) and mounted on a plate by application of freezing (−8 °C) polypropylene glycol for physical support. The leaves were then cut to 30 μm sections using a cooled stainless-steel blade (−20 °C), and the sections transferred to pre-cooled 1 mL cryo-containers and stored in a liquid nitrogen vapour-Dewar until analysis (kept at −196 °C).

Soil samples were also collected near *G*. cf. *sericeum* plants. The samples (*n* = 15 soil samples of 100–200 g each) were collected from the mineral soil approximately 10–25 cm depth.

### Chemical analysis of bulk tissue samples and soils

Plant tissue samples (leaves, wood, bark, flowers) for bulk chemical analysis were collected in the field as described above. These samples were dried at 70 °C for five days in a drying oven and subsequently packed for transport to Australia and gamma irradiated at Steritech Pty. Ltd. in Brisbane following Australian Quarantine Regulations. The dehydrated plant tissue samples were subsequently ground and digested using 4 mL HNO_3_ (70%) and 1 mL H_2_O_2_ (30%) in a microwave oven (Milestone Start D) for a 45-minute programme and diluted to 30 mL with ultrapure water (Millipore 18.2 MΩ·cm at 25 °C) before analysis with ICP-AES (Varian Vista Pro II) for Ni, Co, Cr, Cu, Zn, Mn, Fe, Mg, Ca, Na, K, S and P.

Soil sub-samples (~300 mg) were digested using 9 mL 70% HNO_3_ and 3 mL 37% HCl per sample in a digestion microwave (Milestone Start D) for a program of 1.5 hours, and diluted to 45 mL with ultrapure water before analysis to obtain pseudo-total elemental concentrations. Soil pH was obtained in a mixture of 10 g soil and 25 mL water after 2 hr shaking. Exchangeable trace elements were extracted in 0.1 M Sr(NO_3_)_2_ at a soil:solution ratio of 1:4 (10 gram soil with 40 mL solution) and 2 hr shaking time (adapted^61^). As a means of estimating potentially phytoavailable trace elements, the DTPA-extractant was used according to^62^, which was adapted from the original method^63^, with the following modifications: excluding TEA, adjusted at pH 5.3, 5 g soil with 25 mL extractant, and extraction time of one hr. The soil digests/extracts were analysed with ICP-AES (Varian Vista Pro II) for Ni, Co, Cr, Cu, Zn, Mn, Fe, Mg, Ca, Na, K, S and P.

### Scanning electron microscopy with X-ray microanalysis (SEM-EDS)

The freeze-dried leaf sample (24 hours at −60 °C) was sputter-coated with carbon (∼25 nm) and mounted on an aluminium stub. The sample (see Suppl. Figure 1 prior to carbon coating) was then imaged with scanning electron microscopy with X-ray microanalysis (SEM-EDS) on a JEOL JSM-6610 equipped with EDS (Oxford Instruments 50 mm^2^ X-Max SDD X-ray detector). Maps were made at 300–5000x magnification at 5–15 kV with lower accelerator energies for imaging with secondary returns only and higher accelerator energies for imaging at backscatter mode. EDS spot measurements and maps were made using an electron gun accelerator voltage of 15 kV. Count rates were 1500–3000 cps and quantification was achieved with the AZtecEnergy EDS Microanalysis software with C K-line and O K-line included. Actual reported Wt% values can be interpreted as relative proportions only, and not as absolute concentrations, due to the nature of SEM-EDS calibration for biological material^64^.

### X-ray fluorescence microscopy (XFM)

At the XFM beamline at the Australian Synchrotron (AS) the frozen sections were held between two sheets of Kapton thin-film (4 μm) stretched over a plastic U-frame mounted in a cryo-stream (operated at −140 °C). The samples were transferred to the XFM-sample holder while a cooled (−80 °C) aluminium plate was affixed close to the back of the Kapton thin film (4 mm distance to allow for a cooled envelope), until the sample was in place under the cryo-stream. Whole leaves were imaged similarly. Due to the narrowness of the active cone of the cryo-stream (~5 mm at the centroid – but during scanning the sample could move out of this cone into room temperature) only the small leaf cross-sections remained frozen for the duration of the experiment, whereas the periphery of the larger leaf portion was kept cold and hydrated, but re-thawed repeatedly. The latter caused localised tissue degradation and elemental re-distribution in this zone, with movement of solutes towards the margins (as evidenced by the concentration gradient of multiple elements, particularly in the vicinity of the central vein). The elemental maps (Fig. 4) are cropped to exclude this ‘influence-zone’ with the uncropped maps are provided in the Supplementary Data (Suppl. Figure 2). The cropping was based on the interpretation of the Br and Ca maps – representing the most and the least diffusible elements respectively with contrasting distributions of other elements (Suppl Fig. 3). Bromine rather than K was used to trace labile element motion as the higher energy X-ray fluorescence of Br is more penetrating and so does not suffer from absorption within the specimen which can confound interpretation.

The X-ray fluorescence microscopy (XFM) beamline employs an in-vacuum undulator to produce a brilliant X-ray beam. An Si(111) monochromator and a pair of Kirkpatrick-Baez mirrors delivers a monochromatic focused incident beam onto the specimen^65^. The Maia detector uses a large detector array to maximize detected signal and count-rates for efficient imaging. Maia enables high overall count-rates and uses an annular detector geometry, where the beam passes though the detector and strikes the sample at normal incidence^66,67^. This enables a large solid-angle (1.2 steradian) to be achieved in order to either maximize detected signal or to reduce the dose and potential damage to a specimen^68^. Maia is designed for event-mode data acquisition, where each detected X-ray event is recorded, tagged by detector number in the array, position in the scan and other metadata^69^. This approach eliminates readout delays and enables arbitrarily short pixel times (typically down to 0.1 ms) and large pixel count to be achieved for high definition imaging (typically 10–100 M pixels). The frozen-hydrated samples were mapped by sampling at spatial intervals ranging from 2–10 μm and 0.5–5 ms transit time.

### Synchrotron X-ray Absorption Spectroscopy (XAS)

Ni and Co K-edge XAS spectra of the plant tissue samples and standards were recorded in fluorescence mode at the XAS beamline at the Australian Synchrotron (AS). Monochromation was achieved using a liquid nitrogen cooled double-crystal Si(111) monochromator with a beam size of ~0.5 mm in the horizontal and ~0.1 mm in the vertical direction, and harmonic rejection was achieved by the use of a Rh-coated mirror. The fluorescence data was recorded using a Canberra-Eurisys 36-element Ge-detector positioned at 90° to the incident beam, with the sample positioned at 45° to the incident beam. The X-ray beam energy was calibrated simultaneously with data collection using either a Ni or Co metal foil recorded in transmission downstream of the sample, where the first peak of the first derivative was assumed to be 8331.6 or 7709.5 eV, respectively. The data were collected with 6 eV steps over the pre-edge region (from 8120–8320 eV for Ni and 7510–7690 eV for Co) then 0.25 eV steps over the edge region (from 8320–8380 eV for Ni and from 7690–7760 eV for Co) followed by increments in k-space of 0.035 Å^−1^ until k was equal to 12 Å^−1^.

All samples and standards were analysed at between 5 and 15 K using a closed-cycle He cryostat and contained in poly-lactic acid cuvettes covered with Kapton tape. A total of 14 aqueous Ni^2+^ and Co^2+^ standards were prepared by adding ligands in calculated molar excess (1:6) to Ni^2+^ and Co^2+^ to ensure the formation of Ni-ligand and Co-ligand complexes. The selection of the ligands was based on previous reports of Ni and Co complexation in hyperaccumulator plants^31–33,49^. In particular, oxygen-donor ligands such as carboxylic acids have been implicated. We prepared the aqueous standards with Ni(NO_3_)_2_ or Co(NO_3_)_2_ (ACS trace metal grade) respectively in ultrapure water (Millipore) with the following 11 ligands: aqueous, aconitate, malate, malonate, citrate, oxalate, tartrate, succinate, histidine, glutathione, and cysteine. The solutions were diluted to 5 mM [Ni^2+^] before analysis. The pH of the standards was not adjusted, except for the Ni:citrate standards which were prepared at unadjusted pH, and were then adjusted with NaOH to pH 5.5 (to match the normal vacuolar pH of plant cells), and with the exception of histidine and glutathione which were adjusted to pH 8 to allow for de-protonation and hence coordination via N and S donor atoms, respectively. Glycerol was then added to avoid ice crystal formation (final concentration 33 v/v%) to each standard before flash freezing in liquid nitrogen.

In order to assess for radiation-induced photodamage, repeat spectra were collected from the same samples and the spectra compared to determine if the beam had caused a change in speciation. No such changes were observed in this study, indicating the radiation hardness of the standards and samples under these conditions.

XAS data to k = 12 Å^−1^ were also recorded for 6 Ni^2+^ and Co^2+^ standards (citrate (for both 1:1 and 10:1 citrate:Ni ratios), malate, malonate, tartrate, and hexaquo), with a ligand:Ni (and ligand:Co) ratio of 10:1 and with pH adjusted to 5.5 using NaOH.

### Data processing and statistics

The XRF event stream was analysed using the Dynamic Analysis method^70,71^ as implemented in GeoPIXE^72,73^. GeoPIXE provides quantitative first-order self-absorption corrected maps of projected areal elemental density – maps of elemental content. However, for several of the maps presented in this article, areal-mass density corrected maps are more useful, giving some direct indication of the weight fraction (g/g) of the elemental distributions. In order to achieve this, we have used GeoPIXE’s inbuilt ‘flatten to Compton’ function, which divides the elemental maps of areal density by the Compton scatter map. Although the Compton scatter map is not quantitative, it is to a fair approximation proportional to the integral of the density through the specimen, and so can normalise for variations in thickness and density across the surface of the leaves. Without this normalisation, the thicker mid-vein would dominate the foliar map; normalising to density enables an interesting additional insight into elemental distribution.

Data reduction and analysis of the XAS analysis, including calibration, averaging and background subtraction of all spectra and principal component analysis (PCA), target transformation and linear regression analyses of XANES spectra, were performed using the EXAFSPAK software package (G.N. George, Stanford Synchrotron Radiation Lightsource, Menlo Park, CA, USA). PCA, target transformation and linear combination fits of XANES spectra were performed over the Ni and Co K-edges regions (8320–8450, and 7700–7850 eV, respectively).

## Electronic supplementary material

Supplementary Information
