# Supplementary material for: Simultaneous hyperaccumulation of nickel and cobalt in the tree Glochidion cf. sericeum (Phyllanthaceae): elemental distribution and chemical speciation
Source: Sci Rep. 2018 Jun 26;8:9683. doi: 10.1038/s41598-018-26891-7 (PMC6018747; doi:10.1038/s41598-018-26891-7)
Supplement: Supplementary file 1 — Supplementary Information [file 41598_2018_26891_MOESM1_ESM.pdf]

Simultaneous hyperaccumulation of nickel and cobalt in the tree *Glochidion* cf. *sericeum* (Phyllanthaceae): elemental distribution and chemical speciation

Antony van der Ent[1,2*], Rachel Mak[3], Martin D. de Jonge[4], Hugh H. Harris[5*]

[1]Centre for Mined Land Rehabilitation, Sustainable Minerals Institute, The University of Queensland, Australia.

[2]Laboratoire Sols et Environnement, Université de Lorraine, France.

[3]Department of Chemistry, University of Sydney, Australia

[4]Australian Synchrotron, ANSTO, Australia

[5]Department of Chemistry, The University of Adelaide, Australia.

Corresponding authors: a.vanderent@uq.edu.au, hugh.harris@adelaide.edu.au

SUPPL FIG 1

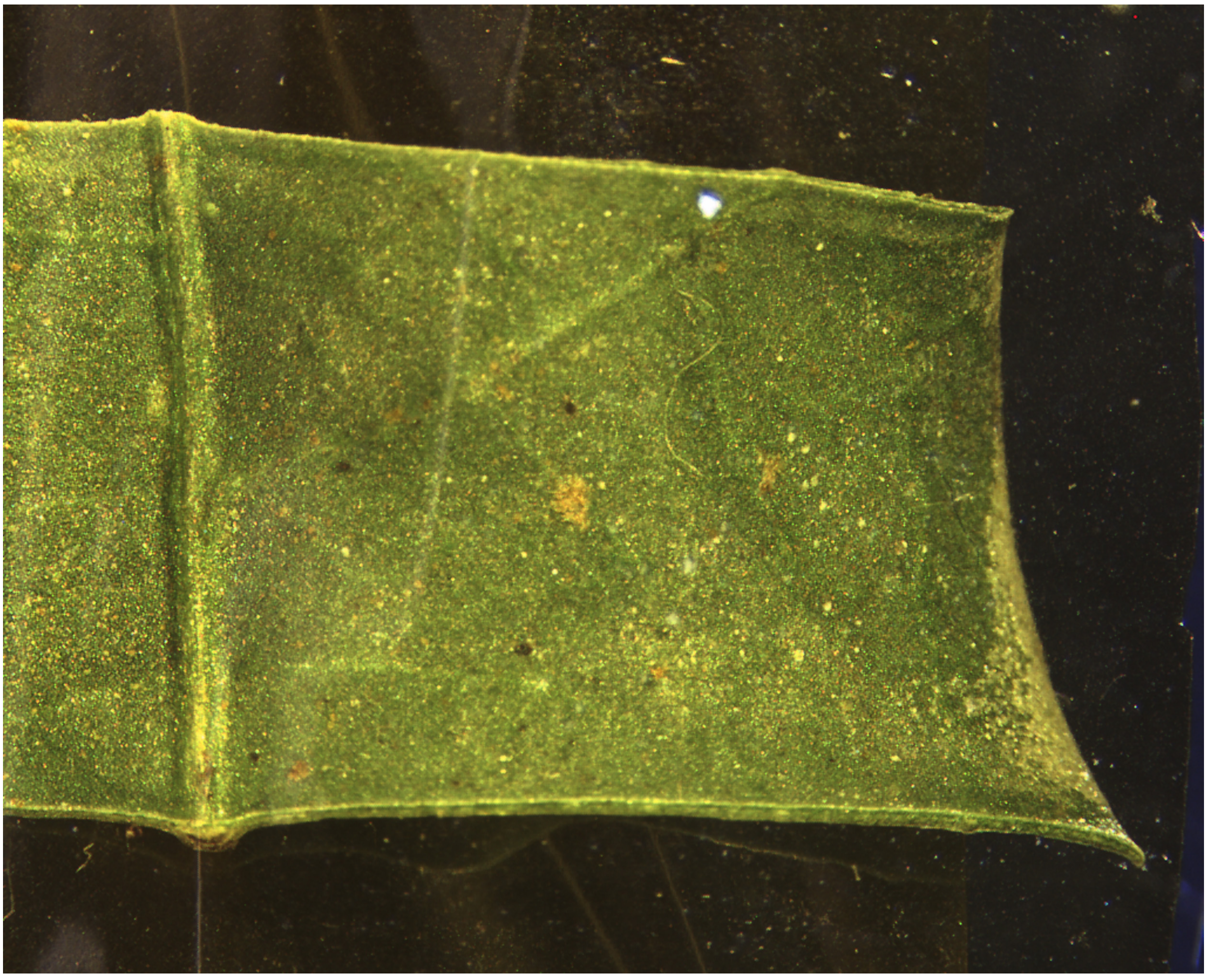

Supplementary Figure 1. Bright-field light microscopy image of the *Glochidion sericeum leaf* from Figures 3–4.

SUPPL FIG 2

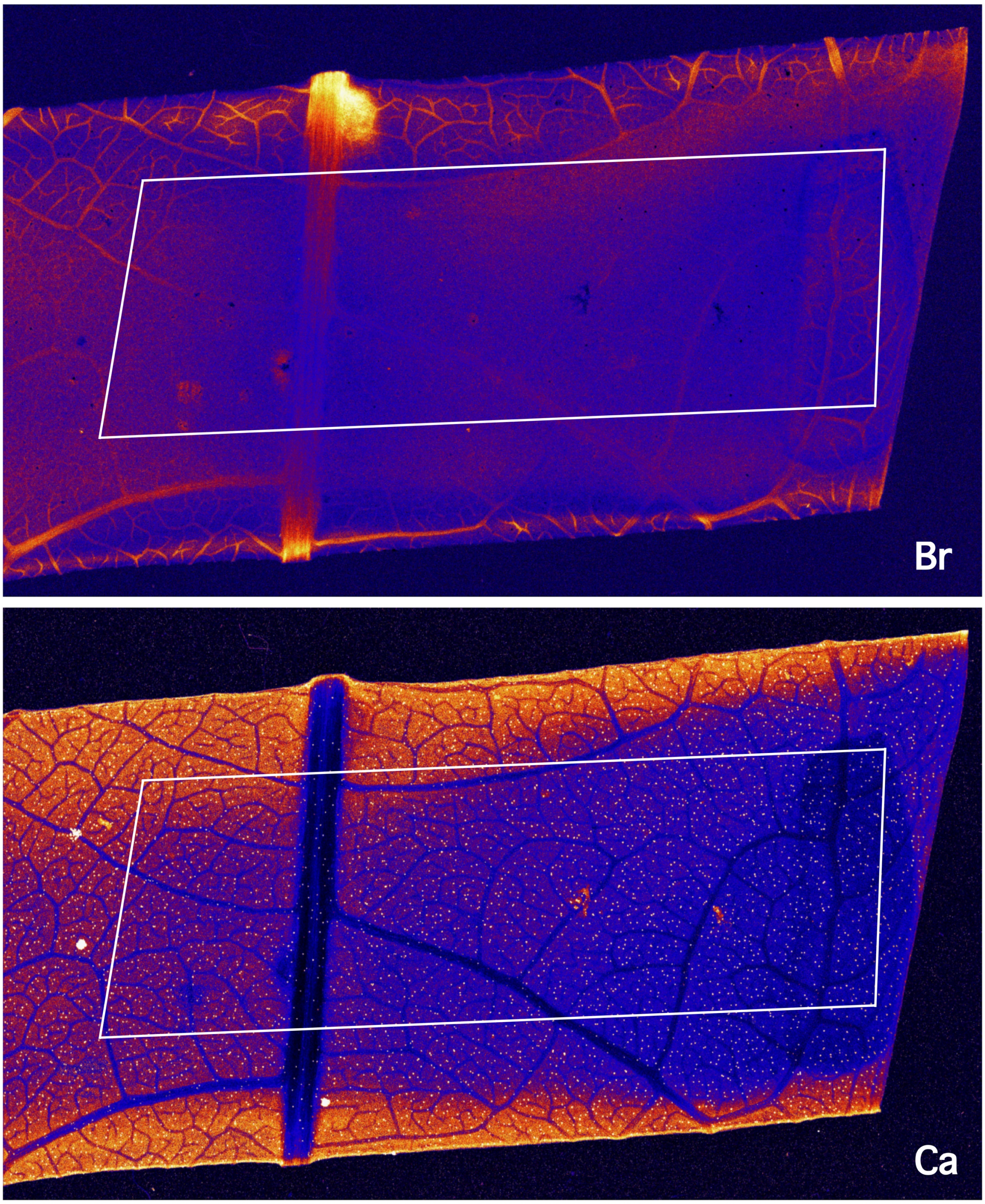

Supplementary Figure 2. Individual elemental µXRF maps for Br and Ca of the frozen-hydrated *Glochidion sericeum* foliar portion from Fig. 3–4. These elements represent the most and the least diffusible elements and were used to probe for the effects of thawing during the XFM scan.

SUPPL FIG 3

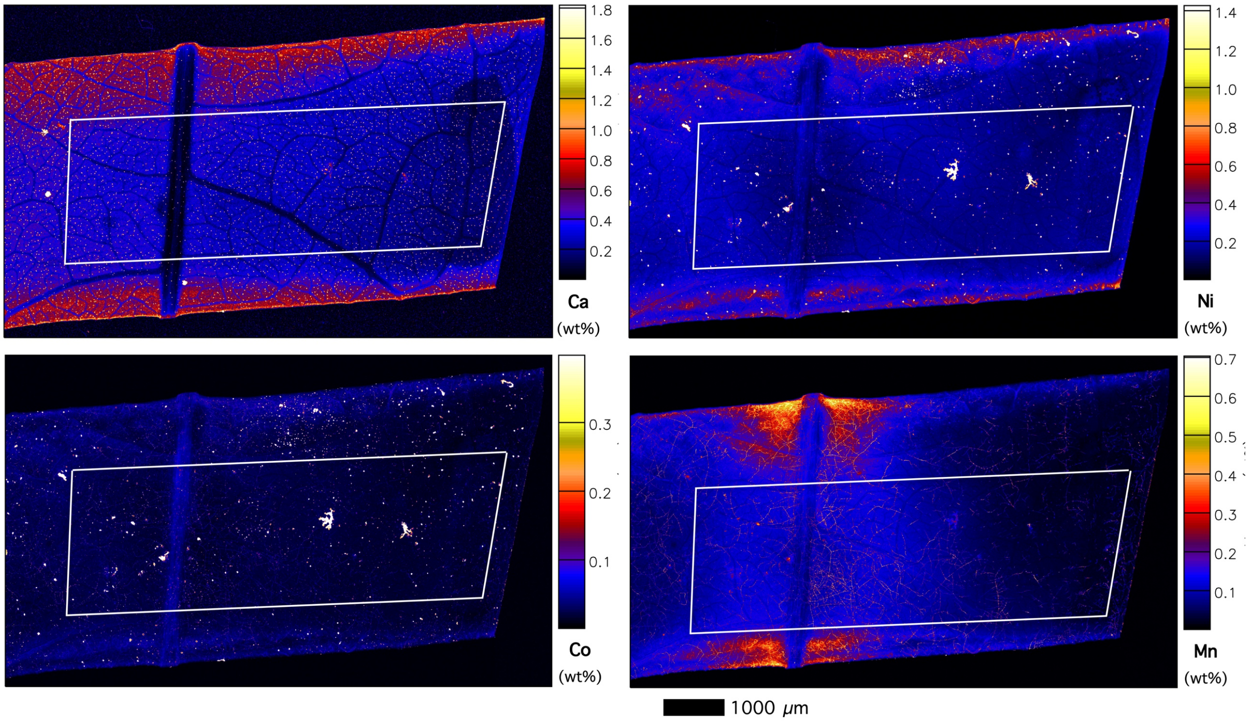

Supplementary Figure 3. Individual elemental μXRF maps of the same frozen-hydrated *Glochidion sericeum* foliar portion from Fig. 3–4 showing K, Ca, Mn, Ni, Zn and Br maps. Unclipped version of file, with the portion used for Fig. 3–4 outlined in white box. Scale bar 1000 μm.

SUPPL FIG 4

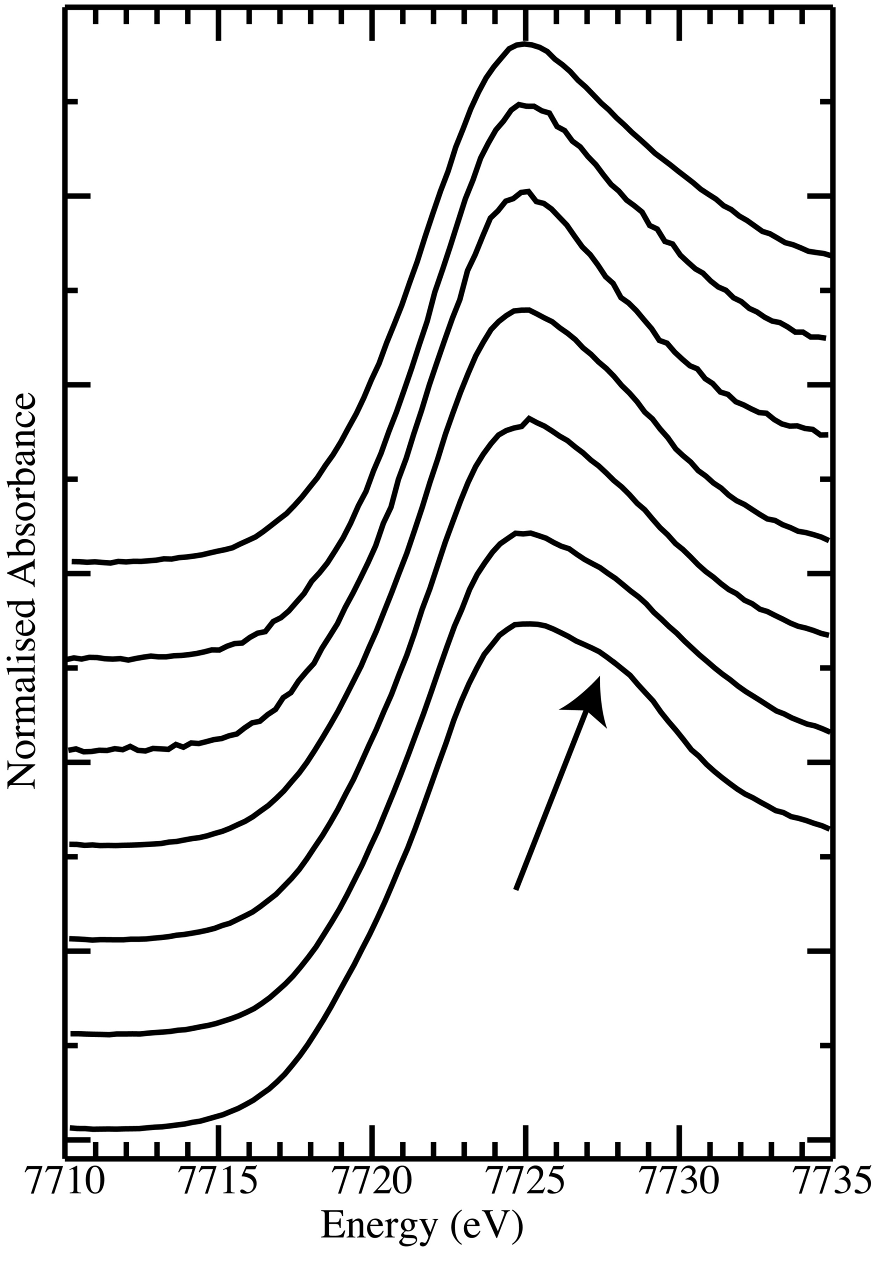

Supplementary Figure 4. Co K-edge X-ray spectra of different sample of *G. sericeum* tissues. The arrow shows a broadening of the peak in some cases with a shoulder occasionally evident on the high-energy side of the peak.

SUPPL FIG 5

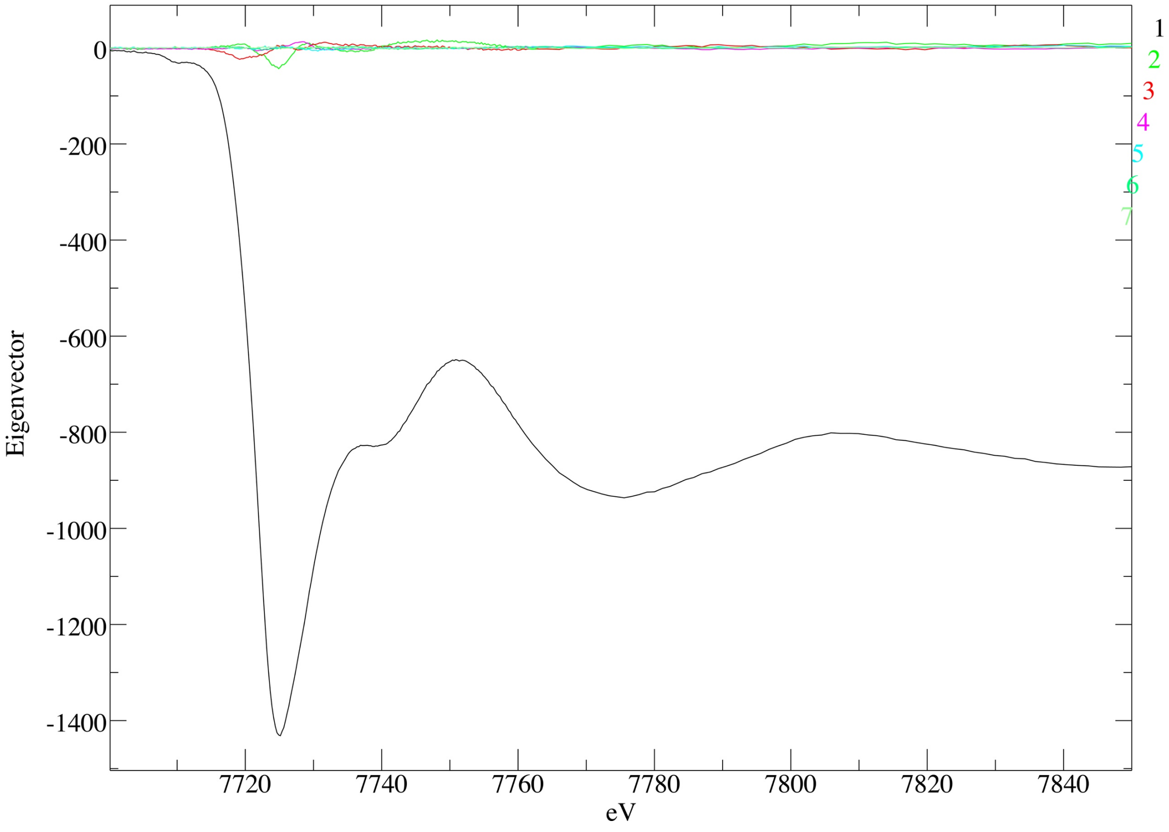

Supplementary Figure 5. Principal Component Analysis of Co K-edge X-ray spectra of different samples of *G. sericeum* leaves and other tissues show in Fig 8.

SUPPL FIG 6

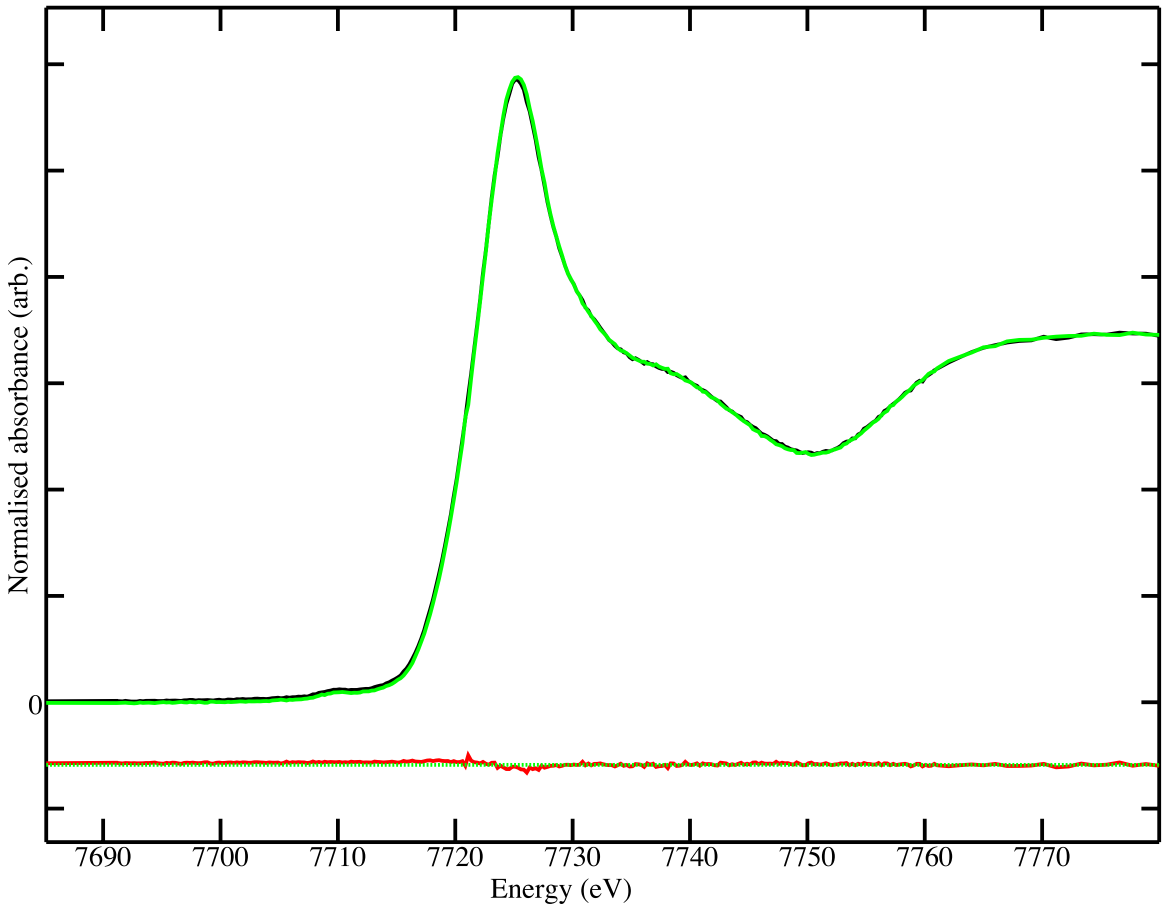

Supplementary Figure 6. Comparison of Co K-edge X-ray spectra. Co:tartrate is the black trace, Co:malate is the green trace and the residual is in red.

SUPPL FIG 7

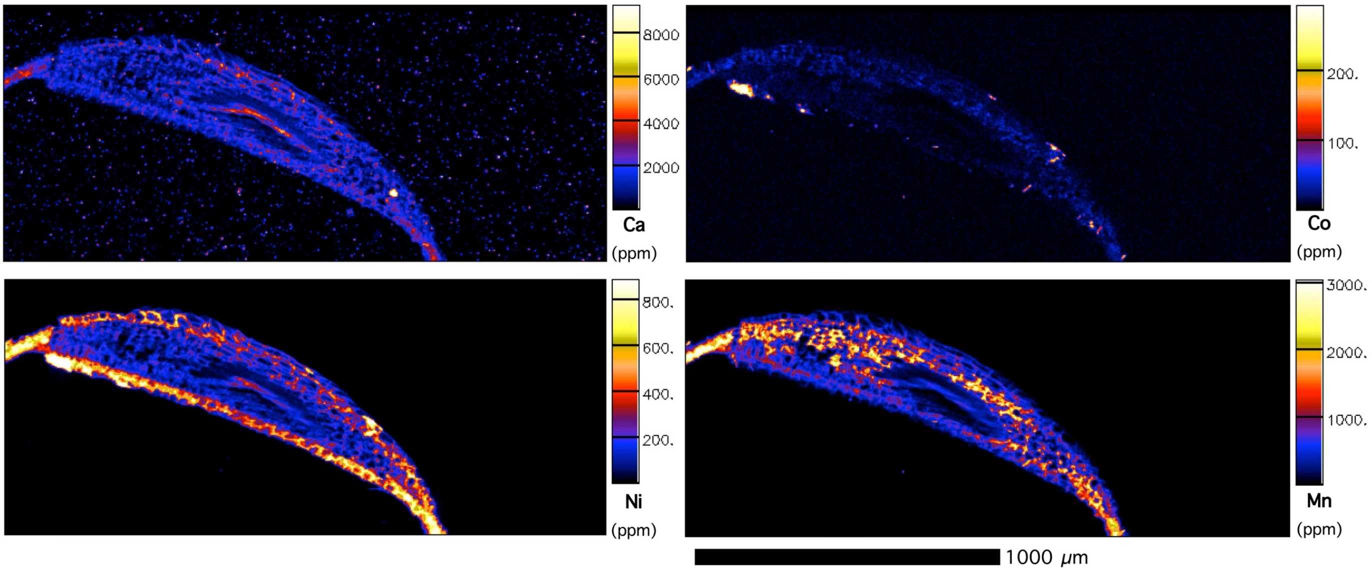

Supplementary Figure 7. µXRF image of a frozen-hydrated *Glochidion sericeum* leaf cross-section displaying elemental maps for Ca, Ni, Co and Mn. Area: 2.00 × 0.85 mm, with a step size of 1 µm and a per-pixel dwell of 1.30 ms. The maps were flattened to Compton map to correct for differences in sample thickness and density.

SUPPL TABLE 1

Herbarium XRF scanning data of *Glochidion* specimen collections in the Forest Research Centre Herbarium in Sepilok.

Specimen herbarium reference	Co [XRF] mg kg^{-1}	Co [corrected] mg kg^{-1}	Ni XRF mg kg^{-1}	Ni [corrected] mg kg^{-1}	Genus	Species	Collector	Date	Area	Locality
SAN84627	<LOD	-	<LOD	-	*Glochidion*	*andersonii*	Diwol S	26/1/77	Papar	Benoni
SAN102559	<LOD	-	<LOD	-	*Glochidion*	*andersonii*	Amin AAS	25/4/86	Beaufort	Binsulok FR
SAN139006	<LOD	-	<LOD	-	*Glochidion*	*andersonii*	Clement M	7/12/93	Beluran	Telupid
SAN139534	44	-	<LOD	-	*Glochidion*	*angulatum*	Asik M	23/8/94	Nabawan	Pensiangan
SAN90830	47	-	<LOD	-	*Glochidion*	*angulatum*	Madani L	9/6/79	Tenom	Tenom
SAN153690	<LOD	-	8309	9943	*Glochidion*	*angulatum*	Suzana S	2/4/13	Tongod	GnTinkar FR
SAN117350	<LOD	-	<LOD	-	*Glochidion*	*angulatum*	Gambating A	6/2/87	Ranau	Ranau
SAN123480	<LOD	-	<LOD	-	*Glochidion*	*angulatum*	Gambating A	19/9/88	Ranau	Ranau
SAN77139	<LOD	-	<LOD	-	*Glochidion*	*angulatum*	Shea G	23/5/73	Ranau	Ranau
SAN114450	<LOD	-	<LOD	-	*Glochidion*	*angulatum*	Madani L	18/6/86	Nabawan	Sapulut
SAN111670	<LOD	-	<LOD	-	*Glochidion*	*angulatum*	Gambating A	4/12/85	Kinabatangan	Tangkulap
SAN136910	33	-	<LOD	-	*Glochidion*	*arborescens*	Sumbing J	24/1/94	Nabawan	Pensiangan FR
SAN28641	44	-	<LOD	-	*Glochidion*	*arborescens*	Bakar AH	8/12/61	Tawau	Quoin Hill FR
SAN115885	52	-	<LOD	-	*Glochidion*	*arborescens*	Fidilis K	29/5/86	Nabawan	Nabawan
SAN121226	63	-	<LOD	-	*Glochidion*	*arborescens*	Majawat G	10/5/87	Pitas	Paitan FR
SAN29575	90	-	<LOD	-	*Glochidion*	*arborescens*	Aban G	11/4/62	Tawau	Apas
SAN27379	96	-	<LOD	-	*Glochidion*	*arborescens*	Buntar A	25/10/61	Tenom	Mt. Mandalom FR
SAN107622	108	-	<LOD	-	*Glochidion*	*arborescens*	Madani L	13/12/84	Beluran	Telupid
SAN107592	121	-	204	-	*Glochidion*	*arborescens*	Madani L	7/12/84	Beluran	Telupid

35068	223	241	<LOD	-	*Glochidion*	*arborescens*	Madani L	21/3/63	Nabawan	Pensiangan
SAN113361	<LOD	-	<LOD	-	*Glochidion*	*arborescens*	Sumbing J	25/1/86	Keningau	Keningau
SAN83103	<LOD	-	<LOD	-	*Glochidion*	*arborescens*	Lamb A	11/5/76	Kinabatangan	Lamag
SAN95629	<LOD	-	<LOD	-	*Glochidion*	*arborescens*	Fidilis K	17/1/83	Tawau	Luasong
SAN96869	<LOD	-	<LOD	-	*Glochidion*	*arborescens*	Lee YF	5/9/83	Sipitang	Maligan
SAN77085	<LOD	-	<LOD	-	*Glochidion*	*arborescens*	Free M	18/10/73	Tawau	Merutai
SAN129968	<LOD	-	<LOD	-	*Glochidion*	*arborescens*	Fidilis K	21/5/92	Nabawan	Milian Labau FR
35074	<LOD	-	<LOD	-	*Glochidion*	*arborescens*	Madani L	21/3/63	Nabawan	Pensiangan
SAN67244	<LOD	-	<LOD	-	*Glochidion*	*arborescens*	Sigin G	29/8/84	Kinabatangan	Pinangah
SAN29466	<LOD	-	<LOD	-	*Glochidion*	*arborescens*	Meijer W	30/3/62	Tawau	Quoin Hill FR
SAN141960	<LOD	-	<LOD	-	*Glochidion*	*arborescens*	Kulip J	21/6/97	Lahad Datu	Tabin FR
SAN30098	<LOD	-	<LOD	-	*Glochidion*	*arborescens*	Singh J	23/5/62	Tawau	Tawau
SAN103218	<LOD	-	<LOD	-	*Glochidion*	*arborescens*	Amin AAS	25/3/87	Beaufort	Weston
39066	45	-	<LOD	-	*Glochidion*	*borneense*	Arshid T	25/11/64	Sandakan	Sandakan
46526	287	263	177	-	*Glochidion*	*borneense*	Mikil G	9/8/64	Ranau	Ranau
SAN115430	<LOD	-	<LOD	-	*Glochidion*	*borneense*	Amin AAS	15/6/88	Beaufort	Beaufort
SAN123524	<LOD	-	<LOD	-	*Glochidion*	*borneense*	Gambating A	3/9/88	Ranau	Mesilau
38344	<LOD	-	<LOD	-	*Glochidion*	*borneense*	Sinanggul HT	11/7/63	Ranau	Ranau
SAN23008	<LOD	-	<LOD	-	*Glochidion*	*borneense*	Meijer W	20/10/60	Kinabatangan	Sg Kapur FR
56923	<LOD	-	<LOD	-	*Glochidion*	*borneense*	Meijer W	23/1/67	Lahad Datu	Silam
SAN69463	43	-	<LOD	-	*Glochidion*	*brunneum*	Amin AAS	12/1/85	Beluran	Beluran
SAN84573	<LOD	-	<LOD	-	*Glochidion*	*brunneum*	Talib B	24/11/76	Beaufort	Bukau
SAN84502	<LOD	-	<LOD	-	*Glochidion*	*brunneum*	Talib B	20/10/76	Beaufort	Lumat
SAN80684	<LOD	-	<LOD	-	*Glochidion*	*brunneum*	Talib B	21/7/76	Papar	Mandahan
SAN129536	<LOD	-	<LOD	-	*Glochidion*	*brunneum*	Diwol S	15/1/90	Lahad Datu	Tabin FR
SAN107345	<LOD	-	<LOD	-	*Glochidion*	*brunneum*	Gambating A	17/11/84	Beluran	Ulu Sapa Payau FR
SAN124666	62	-	<LOD	-	*Glochidion*	*calospermum*	Diwol S	26/5/88	Kinabatangan	Tangkulap FR

SAN32387	73	-	<LOD	-	*Glochidion*	*calospermum*	Badak P	18/11/62	Ranau	Sosopodon FR
48429	84	-	<LOD	-	*Glochidion*	*calospermum*	Singh J	13/1/65	Sandakan	Beluran
SAN28034	136	-	<LOD	-	*Glochidion*	*calospermum*	Carson GL	1/1/62	Ranau	Ranau
SAN28688	<LOD	-	<LOD	-	*Glochidion*	*calospermum*	Meijer W	12/12/61	Tawau	Balung
SAN102604	<LOD	-	<LOD	-	*Glochidion*	*calospermum*	Amin AAS	19/6/86	Beaufort	Beaufort
SAN131024	<LOD	-	<LOD	-	*Glochidion*	*calospermum*	Kulip J	20/2/91	Beluran	Bidu Bidu FR
SAN137118	<LOD	-	<LOD	-	*Glochidion*	*calospermum*	Kuntil L	4/2/93	Beluran	Bidu Bidu FR
SAN149912	<LOD	-	<LOD	-	*Glochidion*	*calospermum*	Joel D	26/11/08	Beluran	Kuamas FR
SAN152642	<LOD	-	<LOD	-	*Glochidion*	*calospermum*	Sugau JB	5/7/12	Kinabatangan	Kuamut FR
254	<LOD	-	<LOD	-	*Glochidion*	*calospermum*	Ming KJ	26/4/00	Kinabatangan	MaliauBasin
SAN116401	<LOD	-	<LOD	-	*Glochidion*	*calospermum*	Gambating A	15/10/86	Ranau	Ranau
SAN105316	<LOD	-	<LOD	-	*Glochidion*	*calospermum*	Fidilis K	14/7/84	Nabawan	Sapulut
33720	<LOD	-	<LOD	-	*Glochidion*	*calospermum*	Putan S	7/1/63	Ranau	Sosopodon FR
77073	<LOD	-	<LOD	-	*Glochidion*	*calospermum*	Free M	11/10/73	Tawau	Tawau
SAN124776	<LOD	-	<LOD	-	*Glochidion*	*calospermum*	Kulip J	19/2/90	Ranau	Trus Madi FR
SAN79118	<LOD	-	<LOD	-	*Glochidion*	*calospermum*	Free M	14/8/75	Kinabatangan	Ulu Segama FR
SNP 27152	1059	523	3754	5053	*Glochidion*	cf. *sericeum*	Van der Ent A	24/3/10	Kinabalu Park	Serinsim
SNP 27152	1087	532	3942	5255	*Glochidion*	cf. *sericeum*	Van der Ent A	24/3/10	Kinabalu Park	Serinsim
SNP 27152	1137	549	3259	4521	*Glochidion*	cf. *sericeum*	Van der Ent A	24/3/10	Kinabalu Park	Serinsim
SNP 27152	1143	551	2046	3219	*Glochidion*	cf. *sericeum*	Van der Ent A	24/3/10	Kinabalu Park	Serinsim
SNP 27152	1202	571	2015	3185	*Glochidion*	cf. *sericeum*	Van der Ent A	24/3/10	Kinabalu Park	Serinsim
SNP 27152	1351	621	2398	3597	*Glochidion*	cf. *sericeum*	Van der Ent A	24/3/10	Kinabalu Park	Serinsim
SNP 27152	1363	625	2479	3683	*Glochidion*	cf. *sericeum*	Van der Ent A	24/3/10	Kinabalu Park	Serinsim
SNP 27152	1554	690	2679	3898	*Glochidion*	cf. *sericeum*	Van der Ent A	24/3/10	Kinabalu Park	Serinsim
SNP 27152	1575	697	2824	4054	*Glochidion*	cf. *sericeum*	Van der Ent A	24/3/10	Kinabalu Park	Serinsim
SNP 27152	1604	706	3042	4288	*Glochidion*	cf. *sericeum*	Van der Ent A	24/3/10	Kinabalu Park	Serinsim
SNP 27152	1604	706	2799	4027	*Glochidion*	cf. *sericeum*	Van der Ent A	24/3/10	Kinabalu Park	Serinsim

SNP 27152	1641	719	2994	4237	*Glochidion*	cf. *sericeum*	Van der Ent A	24/3/10	Kinabalu Park	Serinsim
SNP 27152	1715	744	3307	4572	*Glochidion*	cf. *sericeum*	Van der Ent A	24/3/10	Kinabalu Park	Serinsim
SNP 27152	1721	746	3116	4368	*Glochidion*	cf. *sericeum*	Van der Ent A	24/3/10	Kinabalu Park	Serinsim
SNP 27152	1792	770	3269	4532	*Glochidion*	cf. *sericeum*	Van der Ent A	24/3/10	Kinabalu Park	Serinsim
SNP 27144	1821	779	2858	4090	*Glochidion*	cf. *sericeum*	Van der Ent A	24/3/10	Kinabalu Park	Serinsim
SNP 27152	1942	820	3564	4848	*Glochidion*	cf. *sericeum*	Van der Ent A	24/3/10	Kinabalu Park	Serinsim
SNP 27152	2092	871	7025	8564	*Glochidion*	cf. *sericeum*	Van der Ent A	24/3/10	Kinabalu Park	Serinsim
SNP 27152	2329	951	4003	5320	*Glochidion*	cf. *sericeum*	Van der Ent A	24/3/10	Kinabalu Park	Serinsim
SAN85091	43	-	<LOD	-	*Glochidion*	*elmeri*	Cockburn PF	26/8/76	Lahad Datu	Danum Valley
SAN116799	57	-	61	-	*Glochidion*	*elmeri*	Mansus S	23/6/86	Beluran	Sg Sap iFR
SAN99718	62	-	582	1647	*Glochidion*	*elmeri*	Sigin G	26/7/83	Kinabatangan	Maliau Basin
SAN97302	66	-	<LOD	-	*Glochidion*	*elmeri*	Diwol S	19/6/83	Kinabatangan	Tongod
SAN110199	66	-	50	-	*Glochidion*	*elmeri*	Sumbing J	18/9/85	Kinabatangan	Pinangah
SAN115826	84	-	<LOD	-	*Glochidion*	*elmeri*	Sumbing J	23/5/86	Nabawan	Nabawan
SAN116203	99	-	<LOD	-	*Glochidion*	*elmeri*	Gambating A	27/8/86	Ranau	Ranau
39922	99	-	<LOD	-	*Glochidion*	*elmeri*	Sinanggul HT	10/10/63	Lahad Datu	Silabukan FR
SAN90479	106	-	<LOD	-	*Glochidion*	*elmeri*	Aban G		Beluran	Beluran
SAN82250	122	-	<LOD	-	*Glochidion*	*elmeri*	Fidilis K	19/3/77	Tawau	Luasong
47741	132	-	<LOD	-	*Glochidion*	*elmeri*	Nordin G	16/11/64	Tawau	Apas
SAN97000	160	-	445	1500	*Glochidion*	*elmeri*	Diwol S	14/6/83	Kinabatangan	Tongod
SAN85706	182	227	<LOD	-	*Glochidion*	*elmeri*	Talip AH	15/6/77	Sandakan	Beluran
SAN97442	202	234	674	1746	*Glochidion*	*elmeri*	Gambating A	29/6/83	Kinabatangan	Lamag
SAN116774	208	236	<LOD	-	*Glochidion*	*elmeri*	Sam PP	18/6/86	Beluran	Lung Manis
SAN97000	229	243	470	1527	*Glochidion*	*elmeri*	Diwol S	14/6/83	Kinabatangan	Tongod
SAN30598	1107	539	232	-	*Glochidion*	*elmeri*	Aban G	31/7/62	Tawau	Kalabakan
SAN108910	<LOD	-	149	-	*Glochidion*	*elmeri*	Madani L	8/7/85	Kinabatangan	Ulu Segama FR
SAN74952	<LOD	-	<LOD	-	*Glochidion*	*elmeri*	Diwol S	11/12/71	Beluran	Beluran

SAN75573	<LOD	-	<LOD	-	*Glochidion*	*elmeri*	Kumin M	13/9/72	Beluran	Beluran
38804	<LOD	-	<LOD	-	*Glochidion*	*elmeri*	Agam A	23/8/63	Beluran	Beluran
jd6335	<LOD	-	<LOD	-	*Glochidion*	*elmeri*	Dransfield J	28/2/86	Lahad Datu	Danum Valley
SAN96713	<LOD	-	<LOD	-	*Glochidion*	*elmeri*	Gambating A	20/5/83	Kinabatangan	Kretam FR
SAN33439	<LOD	-	<LOD	-	*Glochidion*	*elmeri*	Chai M	2/3/63	Lahad Datu	Lahad Datu
SAN28774	<LOD	-	<LOD	-	*Glochidion*	*elmeri*	Lajangah JK	26/1/62	Ranau	Lohan
SAN121302	<LOD	-	<LOD	-	*Glochidion*	*elmeri*	Kalantas A	12/5/87	Pitas	Piyas
SAN102435	<LOD	-	<LOD	-	*Glochidion*	*elmeri*	Majawat G	12/10/88	Beluran	Segaliud Lokan FR
SAN81439	<LOD	-	<LOD	-	*Glochidion*	*elmeri*	Madani L	12/3/75	Beluran	Segaliud Lokan FR
SAN81439	<LOD	-	<LOD	-	*Glochidion*	*elmeri*	Madani L	12/3/75	Beluran	Segaliud Lokan FR
37433	<LOD	-	<LOD	-	*Glochidion*	*elmeri*	Sayu E	22/7/63	Beluran	Sg SapiFR
SAN88299	<LOD	-	<LOD	-	*Glochidion*	*elmeri*	Fidilis K	23/2/78	Tawau	Tawau Hill Park FR
SAN55931	<LOD	-	<LOD	-	*Glochidion*	*elmeri*	Nawi AR	6/12/83	Kinabatangan	Tongod
42911	<LOD	-	51	-	*Glochidion*	*glomerulatum*	Masirum R	18/3/64	Lahad Datu	Silabukan FR
SAN127641	<LOD	-	<LOD	-	*Glochidion*	*glomerulatum*	Asik M	21/6/89	Nabawan	Bkt Pisagan
SAN81278	<LOD	-	<LOD	-	*Glochidion*	*glomerulatum*	Arshid T	18/2/75	Sandakan	Gum-Gum FR
SAN103683	<LOD	-	<LOD	-	*Glochidion*	*glomerulatum*	Matin P	21/5/84	Tawau	Kalabakan
SAN102670	<LOD	-	<LOD	-	*Glochidion*	*glomerulatum*	Amin AAS	19/8/86	Sipitang	Mendolong
SAN121166	<LOD	-	<LOD	-	*Glochidion*	*glomerulatum*	Gambating A	19/11/87	Ranau	Ranau
SAN129111	<LOD	-	<LOD	-	*Glochidion*	*glomerulatum*	Soinin S	27/7/87	Ranau	Ranau
SAN105339	<LOD	-	<LOD	-	*Glochidion*	*glomerulatum*	Sumbing J	18/7/84	Nabawan	Sapulut
SAN116824	<LOD	-	<LOD	-	*Glochidion*	*glomerulatum*	Suin G	25/6/86	Beluran	Sg Sapi FR
SAN140657	<LOD	-	<LOD	-	*Glochidion*	*glomerulatum*	Nasrah Y	26/1/96	Beluran	Sg Sapi FR
SAN115536	<LOD	-	<LOD	-	*Glochidion*	*glomerulatum*	Amin AAS	10/2/88	Beaufort	Siaungau FR
SAN106149	<LOD	-	<LOD	-	*Glochidion*	*glomerulatum*	Amin AAS	23/9/91	Sipitang	Sipitang
SAN81030	<LOD	-	<LOD	-	*Glochidion*	*glomerulatum*	Aban G	10/12/74	Ranau	Tampias
SAN149855	<LOD	-	<LOD	-	*Glochidion*	*glomerulatum*	Ubaldus M	18/11/08	Beluran	TawaiFR

SAN107572	<LOD	-	<LOD	-	*Glochidion*	*glomerulatum*	Madani L	6/12/84	Beluran	Telupid
107572	<LOD	-	<LOD	-	*Glochidion*	*glomerulatum*	Madani L	6/12/84	Beluran	Telupid
SAN120183	<LOD	-	<LOD	-	*Glochidion*	*glomerulatum*	Fidilis K	18/3/87	Tenom	Tenom
SAN66797	<LOD	-	<LOD	-	*Glochidion*	*insigns*	Aban G	20/4/70	Tenom	Tenom
38827	<LOD	-	<LOD	-	*Glochidion*	*kerangae*	Sayu E	1/10/63	Sandakan	BatuSapi
SAN126437	<LOD	-	<LOD	-	*Glochidion*	*kerangae*	Amin AAS	13/3/91	Beaufort	Haligolat
SAN103048	<LOD	-	<LOD	-	*Glochidion*	*kerangae*	Amin AAS	23/2/86	KualaPenyu	Kuala Penyu
SAN126466	<LOD	-	<LOD	-	*Glochidion*	*kerangae*	Amin AAS	23/7/92	KualaPenyu	Kuala Penyu
SAN80421	<LOD	-	<LOD	-	*Glochidion*	*kerangae*	Diwol S	9/3/76	KualaPenyu	Kuala Penyu
SAN74163	<LOD	-	<LOD	-	*Glochidion*	*kerangae*	Kumin M	18/8/71	Sandakan	Leila FR
43353	<LOD	-	<LOD	-	*Glochidion*	*kerangae*	Meijer W	6/1/65	Sandakan	Leila FR
43418	<LOD	-	<LOD	-	*Glochidion*	*kerangae*	Pereira WJ	30/4/64	Sandakan	Leila FR
47345	<LOD	-	<LOD	-	*Glochidion*	*kerangae*	Meijer W	3/5/65	Sandakan	Leila FR
SAN27957	<LOD	-	<LOD	-	*Glochidion*	*kerangae*	Thaufeck MR	28/11/61	Sipitang	Mesapol
SAN105962	<LOD	-	<LOD	-	*Glochidion*	*kerangae*	Amin AAS	18/7/91	Beaufort	Siaungau FR
SAN21804	<LOD	-	<LOD	-	*Glochidion*	*kerangae*	Meijer W	21/6/60	Sipitang	Sipitang
SAN103242	<LOD	-	<LOD	-	*Glochidion*	*kerangae*	Amin AAS	27/3/87	Beaufort	Weston
SAN150270	<LOD	-	<LOD	-	*Glochidion*	*laevigatum*	Suzana S	5/4/13	Tongod	Gn Tinkar FR
55-19	<LOD	-	<LOD	-	*Glochidion*	*laevigatum*	AgriRCTuaran	6/9/65	Tuaran	Mengkabong
SAN71100	45	-	<LOD	-	*Glochidion*	*lanceifolium*	Kumin M	25/7/70	Lahad Datu	Lahad Datu
SAN35905	91	-	<LOD	-	*Glochidion*	*lanceifolium*	Aban G	26/4/63	Tawau	Gading
31490	<LOD	-	47	-	*Glochidion*	*lanceifolium*	Agam A	19/5/63	Lahad Datu	Silam
771987	<LOD	-	63	-	*Glochidion*	*lanceifolium*	Argent G	12/3/87	Lahad Datu	Danum Valley
SAN111026	<LOD	-	<LOD	-	*Glochidion*	*lanceifolium*	Gambating A	27/8/85	Beluran	Beluran
SAN91100	<LOD	-	<LOD	-	*Glochidion*	*lanceifolium*	Diwol S	14/12/79	Beluran	Beluran
111026	<LOD	-	<LOD	-	*Glochidion*	*lanceifolium*	Gambating A	27/8/85	Beluran	Beluran
SAN108117	<LOD	-	<LOD	-	*Glochidion*	*lanceifolium*	Gambating A	18/2/85	Sandakan	Gomantong Caves

SAN64847	<LOD	-	<LOD	-	*Glochidion*	*lanceifolium*	Aban G	21/11/83	Nabawan	Nabawan
SAN110280	<LOD	-	<LOD	-	*Glochidion*	*lanceifolium*	Asik M	25/9/85	Kinabatangan	Pinangah
55351	<LOD	-	<LOD	-	*Glochidion*	*lanceifolium*	Aban G	4/4/66	Lahad Datu	Silam
35010	<LOD	-	<LOD	-	*Glochidion*	*lanceilimbum*	Madani L	9/3/63	Keningau	Keningau
33680	<LOD	-	<LOD	-	*Glochidion*	*lanceilimbum*	Mujin MA	31/3/63	KotaBelud	Lemaas FR
55723	<LOD	-	<LOD	-	*Glochidion*	*lanceilimbum*	Binideh RP	29/4/68	Tenom	Tomani
SAN96827	52	-	<LOD	-	*Glochidion*	*lanceisepalum*	Aban G	27/5/83	Sandakan	Sepilok Kabili FR
SAN73411	55	-	<LOD	-	*Glochidion*	*lanceisepalum*	Shea G	15/11/71	Keningau	Keningau
52546	68	-	<LOD	-	*Glochidion*	*lanceisepalum*	Talip AH	31/7/65	Lahad Datu	Lahad Datu
SAN32266	<LOD	-	<LOD	-	*Glochidion*	*lanceisepalum*	Lajangah JK	18/10/62	Beaufort	Beaufort
SAN112326	<LOD	-	<LOD	-	*Glochidion*	*lanceisepalum*	Campbell EJF	22/6/86	Lahad Datu	Danum Valley
SAN123723	<LOD	-	<LOD	-	*Glochidion*	*lanceisepalum*	Majawat G	14/3/88	Lahad Datu	Danum Valley
SAN82393	<LOD	-	<LOD	-	*Glochidion*	*lanceisepalum*	Saikeh L	20/10/75	Kudat	Kudat
SAN108228	<LOD	-	<LOD	-	*Glochidion*	*lanceisepalum*	Argent G	25/2/85	Lahad Datu	Lahad Datu
SAN118459	<LOD	-	<LOD	-	*Glochidion*	*lanceisepalum*	Fidilis K	22/10/86	Keningau	Lanas
SAN89737	<LOD	-	<LOD	-	*Glochidion*	*lanceisepalum*	Fidilis K	22/2/79	Tawau	Luasong
SAN104303	<LOD	-	<LOD	-	*Glochidion*	*lanceisepalum*	Fidilis K	18/5/87	Nabawan	Nabawan
SAN83840	<LOD	-	<LOD	-	*Glochidion*	*lanceisepalum*	Diwol S	14/8/76	Nabawan	Nabawan
SAN109244	<LOD	-	<LOD	-	*Glochidion*	*lanceisepalum*	Mansus S	6/6/85	Kinabatangan	Pinangah
SAN116847	<LOD	-	<LOD	-	*Glochidion*	*lanceisepalum*	Martin G	30/6/86	Beluran	Segaliud Lokan FR
SAN85364	<LOD	-	<LOD	-	*Glochidion*	*lanceisepalum*	Fidilis K	11/2/77	Tawau	Tawau
SAN108884	<LOD	-	<LOD	-	*Glochidion*	*lanceisepalum*	Madani L	15/4/85	Kinabatangan	Ulu Segama FR
SAN95270	<LOD	-	<LOD	-	*Glochidion*	*lanceisepalum*	Gambating A	5/4/83	Ranau	Ulu Tungud FR
36-37	37	-	<LOD	-	*Glochidion*	*littorale*	AgriRCTuaran	6/9/65	Tuaran	Mengkabong
SAN107450	45	-	<LOD	-	*Glochidion*	*littorale*	Ismail AAP	16/11/84	Beluran	Ulu Sg Muanod
59-9	52	-	<LOD	-	*Glochidion*	*littorale*	AgriRCTuaran	6/9/65	Tuaran	Mengkabong
59-12	52	-	<LOD	-	*Glochidion*	*littorale*	AgriRCTuaran	6/9/65	Tuaran	Mengkabong

SAN109951	57	-	<LOD	-	*Glochidion*	*littorale*	Asik M	9/8/85	Nabawan	Nabawan
SAN123464	64	-	107	-	*Glochidion*	*littorale*	Gambating A	12/9/88	Ranau	Mesilau
SAN102148	<LOD	-	<LOD	-	*Glochidion*	*littorale*	Lee YF	6/10/84	Beluran	Beluran
SAN84629	<LOD	-	<LOD	-	*Glochidion*	*littorale*	Diwol S	26/1/77	Papar	Benoni
SAN98771	<LOD	-	<LOD	-	*Glochidion*	*littorale*	Amin AAS	22/7/93	Beaufort	Binsulok
SAN104141	<LOD	-	<LOD	-	*Glochidion*	*littorale*	Amin AAS	20/2/92	Beaufort	Binsulok FR
SAN114762	<LOD	-	<LOD	-	*Glochidion*	*littorale*	Amin AAS	15/1/88	Beaufort	Binsulok FR
SAN35488	<LOD	-	<LOD	-	*Glochidion*	*littorale*	Ahwing J	9/4/63	Kinabatangan	Dagat
SAN88486	<LOD	-	<LOD	-	*Glochidion*	*littorale*	Fidilis K	27/9/78	Tawau	Kalabakan
SAN103445	<LOD	-	<LOD	-	*Glochidion*	*littorale*	Amin AAS	17/6/87	KualaPenyu	Kuala Penyu
SAN114971	<LOD	-	<LOD	-	*Glochidion*	*littorale*	Amin AAS	28/2/88	KualaPenyu	Kuala Penyu
SAN126491	<LOD	-	<LOD	-	*Glochidion*	*littorale*	Amin AAS	27/7/92	KualaPenyu	Kuala Penyu
SAN66262	<LOD	-	<LOD	-	*Glochidion*	*littorale*	Cockburn PF	11/6/69	KualaPenyu	Kuala Penyu
35782	<LOD	-	<LOD	-	*Glochidion*	*littorale*	Mikil G	1/4/63	Sandakan	Labuk Road FR
SAN94431	<LOD	-	<LOD	-	*Glochidion*	*littorale*	Gambating A	22/4/82	Sandakan	Leila FR
SAN96274	<LOD	-	<LOD	-	*Glochidion*	*littorale*	Aban G	16/9/83	Sandakan	Leila FR
61731	<LOD	-	<LOD	-	*Glochidion*	*littorale*	Binson S	7/2/68	Sandakan	Leila FR
52632	<LOD	-	<LOD	-	*Glochidion*	*littorale*	Kanis A	8/10/65	Sandakan	Leila FR
SAN32645	<LOD	-	<LOD	-	*Glochidion*	*littorale*	Jawanting A	24/11/62	Beluran	Mamahat FR
SAN103092	<LOD	-	<LOD	-	*Glochidion*	*littorale*	Amin AAS	15/4/86	Papar	Mandahan
SAN122849	<LOD	-	<LOD	-	*Glochidion*	*littorale*	Amin AAS	29/6/89	Papar	Mandahan
SAN126592	<LOD	-	<LOD	-	*Glochidion*	*littorale*	Amin AAS	27/4/92	Papar	Mandahan
SAN32239	<LOD	-	<LOD	-	*Glochidion*	*littorale*	Talip APA	27/10/62	Papar	Mandahan
SAN78046	<LOD	-	<LOD	-	*Glochidion*	*littorale*	Diwol S	25/3/74	Papar	Mandahan
SAN80013	<LOD	-	<LOD	-	*Glochidion*	*littorale*	Diwol S	5/6/75	Papar	Mandahan
47880	<LOD	-	<LOD	-	*Glochidion*	*littorale*	Sinanggul HT	27/10/64	Papar	Mandahan
SAN102637	<LOD	-	<LOD	-	*Glochidion*	*littorale*	Amin AAS	12/7/86	Sipitang	Marintaman

SAN102923	<LOD	-	<LOD	-	*Glochidion*	*littorale*	Amin AAS	23/10/85	Beaufort	Membakut
SAN102928	<LOD	-	<LOD	-	*Glochidion*	*littorale*	Amin AAS	25/10/85	Beaufort	Membakut
SAN111356	<LOD	-	<LOD	-	*Glochidion*	*littorale*	Madani L	10/9/85	Beaufort	Membakut
SAN115227	<LOD	-	<LOD	-	*Glochidion*	*littorale*	Amin AAS	28/8/88	Beaufort	Membakut
SAN115351	<LOD	-	<LOD	-	*Glochidion*	*littorale*	Amin AAS	20/1/90	Beaufort	Membakut
SAN126140	<LOD	-	<LOD	-	*Glochidion*	*littorale*	Amin AAS	28/1/91	Beaufort	Membakut
SAN106009	<LOD	-	<LOD	-	*Glochidion*	*littorale*	Amin AAS	21/8/91	Sipitang	Mengalong FR
SAN115080	<LOD	-	<LOD	-	*Glochidion*	*littorale*	Amin AAS	22/9/87	Sipitang	Mengalong FR
SAN84360	<LOD	-	<LOD	-	*Glochidion*	*littorale*	Talib B	16/8/76	Sipitang	Mengalong FR
44-54	<LOD	-	<LOD	-	*Glochidion*	*littorale*	AgriRCTuaran	6/9/65	Tuaran	Mengkabong
55-17	<LOD	-	<LOD	-	*Glochidion*	*littorale*	AgriRCTuaran	6/9/65	Tuaran	Mengkabong
SAN86793	<LOD	-	<LOD	-	*Glochidion*	*littorale*	Stone BC	12/4/77	Kudat	Pulau Balambangan
SAN87077	<LOD	-	<LOD	-	*Glochidion*	*littorale*	Stone BC	7/4/77	Kudat	Pulau Balambangan
SAN87096	<LOD	-	<LOD	-	*Glochidion*	*littorale*	Stone BC	8/4/77	Kudat	Pulau Balambangan
SAN149628	<LOD	-	<LOD	-	*Glochidion*	*littorale*	Ubaldus M	25/7/08	Tawau	Pulau Bingluran
SAN136213	<LOD	-	<LOD	-	*Glochidion*	*littorale*	Meijer W	30/6/92	KotaKinabalau	Pulau Gaya
56129	<LOD	-	<LOD	-	*Glochidion*	*littorale*	Kanis A	17/2/66	KotaKinabalu	Pulau Gaya
33697	<LOD	-	<LOD	-	*Glochidion*	*littorale*	Chong J	6/4/63	KotaKinabalu	Pulau Gaya
SAN136587	<LOD	-	<LOD	-	*Glochidion*	*littorale*	Meijer W	8/8/92	KotaKinabalu	Pulau Manukan
SAN103197	<LOD	-	<LOD	-	*Glochidion*	*littorale*	Amin AAS	16/3/87	KualaPenyu	Pulau Tiga
SAN126034	<LOD	-	<LOD	-	*Glochidion*	*littorale*	Amin AAS	15/3/90	KualaPenyu	Pulau Tiga
SAN126315	<LOD	-	<LOD	-	*Glochidion*	*littorale*	Amin AAS	23/5/92	KualaPenyu	Pulau Tiga
SAN126833	<LOD	-	<LOD	-	*Glochidion*	*littorale*	Amin AAS	9/3/89	KualaPenyu	Pulau Tiga
SAN126926	<LOD	-	<LOD	-	*Glochidion*	*littorale*	Amin AAS	20/5/91	KualaPenyu	Pulau Tiga
SAN127172	<LOD	-	<LOD	-	*Glochidion*	*littorale*	Amin AAS	26/4/93	KualaPenyu	Pulau Tiga
SAN86546	<LOD	-	<LOD	-	*Glochidion*	*littorale*	Amin AAS	13/8/85	KualaPenyu	Pulau Tiga
SAN122301	<LOD	-	<LOD	-	*Glochidion*	*littorale*	Meijer W	15/9/87	Sandakan	Sandakan

SAN107858	<LOD	-	<LOD	-	*Glochidion*	*littorale*	Gambating A	15/5/85	Sandakan	Sepilok Kabili FR
SAN81159	<LOD	-	<LOD	-	*Glochidion*	*littorale*	Saikeh L	11/2/75	Sandakan	Sepilok Kabili FR
SAN115546	<LOD	-	<LOD	-	*Glochidion*	*littorale*	Amin AAS	11/2/88	Beaufort	Siaungau FR
SAN115064	<LOD	-	<LOD	-	*Glochidion*	*littorale*	Amin AAS	22/8/87	Sipitang	Sipitang
SAN108013	<LOD	-	<LOD	-	*Glochidion*	*littorale*	Sigin G	7/12/85	Kinabatangan	Tangkulap FR
SAN149713	<LOD	-	<LOD	-	*Glochidion*	*littorale*	Dauni S	30/8/08	Kinabatangan	Trusan Kinabatangan
SAN67017	<LOD	-	<LOD	-	*Glochidion*	*littorale*	Hepburn AJ	17/11/70	Sipitang	Usok
SAN103070	<LOD	-	<LOD	-	*Glochidion*	*littorale*	Amin AAS	18/3/86	Beaufort	Weston
SAN43213	<LOD	-	<LOD	-	*Glochidion*	*littorale*	Meijer W	28/6/64	Beaufort	Weston
43213	<LOD	-	<LOD	-	*Glochidion*	*littorale*	Meijer W	28/6/64	Beaufort	Weston
SAN154155	38	-	<LOD	-	*Glochidion*	*lutescens*	Pius G	26/10/11	Keningau	Trus Madi
SAN78189	50	-	<LOD	-	*Glochidion*	*lutescens*	Diwol S	19/6/74	Beaufort	Beaufort
44545	65	-	<LOD	-	*Glochidion*	*lutescens*	Lajangah JK	18/7/65	Beaufort	Lumat
49994	68	-	<LOD	-	*Glochidion*	*lutescens*	Meijer W	14/4/65	Lahad Datu	Lahad Datu
SAN150781	75	-	<LOD	-	*Glochidion*	*lutescens*	Pius G	23/3/10	Ranau	Ranau
SAN19020	80	-	<LOD	-	*Glochidion*	*lutescens*	Ahwing J	9/12/62	Sandakan	Sandakan
SAN56029	82	-	<LOD	-	*Glochidion*	*lutescens*	Maikin L	9/5/84	Tambunan	Kirokot
41885	99	-	<LOD	-	*Glochidion*	*lutescens*	Mikil G	14/11/64	Tambunan	Tambunan
SAN82764	239	246	106	-	*Glochidion*	*lutescens*	Saikeh L	26/1/76	Ranau	Kinabalu National Park
SAN70581	<LOD	-	69	-	*Glochidion*	*lutescens*	Kumin M	29/8/70	Kinabatangan	Kuala Karamuak
SAN86671	<LOD	-	<LOD	-	*Glochidion*	*lutescens*	Fidilis K	19/4/77	Tawau	Apas
40215	<LOD	-	<LOD	-	*Glochidion*	*lutescens*	Jawanting A	16/6/64	Beaufort	Beaufort
SAN99222	<LOD	-	<LOD	-	*Glochidion*	*lutescens*	Aban G	14/7/83	Beluran	Beluran
SAN33739	<LOD	-	<LOD	-	*Glochidion*	*lutescens*	Putan S	29/1/63	KotaKinabalu	Bukit Padang
SAN112149	<LOD	-	<LOD	-	*Glochidion*	*lutescens*	Aik J	2/6/86	Lahad Datu	Danum Valley
SAN125532	<LOD	-	<LOD	-	*Glochidion*	*lutescens*	Asik M	19/8/88	Tambunan	Gn Trus Madi

SAN122778	<LOD	-	<LOD	-	*Glochidion*	*lutescens*	Bousi J	21/1/88	Kinabatangan	Gomantong FR
SAN95723	<LOD	-	<LOD	-	*Glochidion*	*lutescens*	Fidilis K	17/3/83	Tawau	Kalabakan
SAN79837	<LOD	-	<LOD	-	*Glochidion*	*lutescens*	Aban G	24/7/74	Kunak	Kalumpang FR
SAN113081	<LOD	-	<LOD	-	*Glochidion*	*lutescens*	Sumbing J	16/11/85	Keningau	Keningau
SAN102944	<LOD	-	<LOD	-	*Glochidion*	*lutescens*	Amin AAS	15/11/85	Beaufort	Kg Hindian FR
SAN84524	<LOD	-	<LOD	-	*Glochidion*	*lutescens*	Talib B	16/11/76	Beaufort	Kg Hindian FR
1252	<LOD	-	<LOD	-	*Glochidion*	*lutescens*	Sato T	16/8/81	Papar	Kimanis
SAN80737	<LOD	-	<LOD	-	*Glochidion*	*lutescens*	Talib B	28/7/76	Beaufort	Klias FR
SAN97065	<LOD	-	<LOD	-	*Glochidion*	*lutescens*	Aban G	8/6/83	Kinabatangan	Kretam
SAN108217	<LOD	-	<LOD	-	*Glochidion*	*lutescens*	Argent G	24/2/85	Lahad Datu	Lahad Datu
SAN117126	<LOD	-	<LOD	-	*Glochidion*	*lutescens*	Kuntil L	24/6/87	Lahad Datu	Lahad Datu
SAN96802	<LOD	-	<LOD	-	*Glochidion*	*lutescens*	Lee YF	6/5/83	Lahad Datu	Lahad Datu
51325	<LOD	-	<LOD	-	*Glochidion*	*lutescens*	Meijer W	11/8/65	Kinabatangan	Lamag
SAN75603	<LOD	-	<LOD	-	*Glochidion*	*lutescens*	Shea G	20/4/72	Tawau	Luasong
SAN102734	<LOD	-	<LOD	-	*Glochidion*	*lutescens*	Amin AAS	23/10/86	Beaufort	Lumat
SAN77898	<LOD	-	<LOD	-	*Glochidion*	*lutescens*	Karim AM	8/9/73	Beaufort	Lumat
SAN69851	<LOD	-	<LOD	-	*Glochidion*	*lutescens*	Lee YF	7/9/83	Sipitang	Maligan
SAN103383	<LOD	-	<LOD	-	*Glochidion*	*lutescens*	Amin AAS	14/4/87	Sipitang	Marintaman
SAN103278	<LOD	-	<LOD	-	*Glochidion*	*lutescens*	Amin AAS	21/11/86	Beaufort	Membakut
SAN106047	<LOD	-	<LOD	-	*Glochidion*	*lutescens*	Amin AAS	25/8/91	Sipitang	Mengalong FR
SAN124146	<LOD	-	<LOD	-	*Glochidion*	*lutescens*	Diwol S	21/6/88	Sipitang	Mengalong FR
SAN82606	<LOD	-	<LOD	-	*Glochidion*	*lutescens*	Madani L	17/11/75	Ranau	Merungin
SAN82676	<LOD	-	<LOD	-	*Glochidion*	*lutescens*	Madani L	22/11/75	Ranau	Merungin
SAN111431	<LOD	-	<LOD	-	*Glochidion*	*lutescens*	Madani L	16/9/85	Sipitang	Mesapol FR
SAN120756	<LOD	-	<LOD	-	*Glochidion*	*lutescens*	Bousi J	31/7/87	Lahad Datu	Mt Silam
SAN80898	<LOD	-	<LOD	-	*Glochidion*	*lutescens*	Diwol S	19/6/76	Nabawan	Nabawan
SAN60468	<LOD	-	<LOD	-	*Glochidion*	*lutescens*	Amin AAS	23/7/84	Penampang	Penampang

SAN100385	<LOD	-	<LOD	-	*Glochidion*	*lutescens*	Rahim A	19/9/83	Kinabatangan	Pinangah
SAN117176	<LOD	-	<LOD	-	*Glochidion*	*lutescens*	Mansus S	23/10/86	Kinabatangan	Pinangah
SAN60234	<LOD	-	<LOD	-	*Glochidion*	*lutescens*	Madani L	24/8/84	Kinabatangan	Pinangah
SAN93034	<LOD	-	<LOD	-	*Glochidion*	*lutescens*	Aban G	11/9/80	Semporna	Pulau Boheydulang
SAN123205	<LOD	-	<LOD	-	*Glochidion*	*lutescens*	Gambating A	16/4/88	Ranau	Ranau
SAN97240	<LOD	-	<LOD	-	*Glochidion*	*lutescens*	Aban G	16/6/83	Sandakan	Sandakan
SAN127075	<LOD	-	<LOD	-	*Glochidion*	*lutescens*	Amin AAS	23/11/90	Beaufort	Siaungau FR
SAN102649	<LOD	-	<LOD	-	*Glochidion*	*lutescens*	Amin AAS	15/7/86	Sipitang	Sipitang
SAN126505	<LOD	-	<LOD	-	*Glochidion*	*lutescens*	Amin AAS	26/5/89	Sipitang	Sipitang
SAN73300	<LOD	-	<LOD	-	*Glochidion*	*lutescens*	Saikeh L	22/5/71	Keningau	Sook
SAN84219	<LOD	-	<LOD	-	*Glochidion*	*lutescens*	Nordin AA	22/1/77	Keningau	Sook
SAN142601	<LOD	-	<LOD	-	*Glochidion*	*lutescens*	Madani L	8/9/98	Penampang	Sugud
SAN124970	<LOD	-	<LOD	-	*Glochidion*	*lutescens*	Soinin S	21/10/89	Tambunan	Tambunan
SAN109641	<LOD	-	<LOD	-	*Glochidion*	*lutescens*	Majawat G	12/5/89	Kinabatangan	Tangkulap FR
SAN141163	<LOD	-	<LOD	-	*Glochidion*	*lutescens*	Goh SH	11/6/98	Tawau	Tawau
SAN133972	<LOD	-	<LOD	-	*Glochidion*	*lutescens*	Madani L	25/8/92	Beluran	Telupid
SAN91266	<LOD	-	<LOD	-	*Glochidion*	*lutescens*	Aban G	17/8/79	Beluran	Telupid
SAN117525	<LOD	-	<LOD	-	*Glochidion*	*lutescens*	Maikin L	29/10/86	Kinabatangan	Tongod
SAN80404	<LOD	-	<LOD	-	*Glochidion*	*lutescens*	Talib B	24/2/76	Keningau	Tulid
SAN84296	<LOD	-	<LOD	-	*Glochidion*	*lutescens*	Nordin AA	11/3/77	Keningau	Tulid
SAN84302	<LOD	-	<LOD	-	*Glochidion*	*lutescens*	Nordin AA	12/3/77	Keningau	Tulid
SAN85818	<LOD	-	<LOD	-	*Glochidion*	*lutescens*	Nordin AA	23/7/77	Keningau	Tulid
SAN99836	<LOD	-	<LOD	-	*Glochidion*	*lutescens*	Rahim A	2/8/83	Beluran	Ulu Tungud FR
SAN103217	<LOD	-	<LOD	-	*Glochidion*	*lutescens*	Amin AAS	25/3/87	Beaufort	Weston
SAN126369	<LOD	-	<LOD	-	*Glochidion*	*macrostigma*	Amin AAS	25/6/92	Beaufort	Beaufort
SAN98777	<LOD	-	<LOD	-	*Glochidion*	*macrostigma*	Amin AAS	23/7/93	Beaufort	Binsulok FR
SAN108151	<LOD	-	<LOD	-	*Glochidion*	*macrostigma*	Gambating A	13/2/85	Kinabatangan	Keruak FR

SAN97333	<LOD	-	<LOD	-	*Glochidion*	*macrostigma*	Diwol S	20/6/83	Kinabatangan	Kuala Karamuak
SAN102773	<LOD	-	<LOD	-	*Glochidion*	*macrostigma*	Amin AAS	14/9/85	Beaufort	Lumat
SAN100066	<LOD	-	<LOD	-	*Glochidion*	*macrostigma*	Aban G	19/8/83	KotaMarudu	Marak Parak
SAN135660	<LOD	-	<LOD	-	*Glochidion*	*macrostigma*	Fidilis K	14/7/93	Kinabatangan	Pinangah
SAN90225	<LOD	-	<LOD	-	*Glochidion*	*macrostigma*	Aban G	2/6/79	Ranau	Ranau
SAN154844	<LOD	-	<LOD	-	*Glochidion*	*macrostigma*	Suzana S	13/6/12	Sapulut	Sg Siliawan
SAN67601	<LOD	-	<LOD	-	*Glochidion*	*macrostigma*	Amin AAS	18/9/84	Kinabatangan	Tongod
SAN108729	<LOD	-	<LOD	-	*Glochidion*	*macrostigma*	Madani L	14/4/85	Kinabatangan	Ulu Segama FR
SAN99783	<LOD	-	<LOD	-	*Glochidion*	*macrostigma*	Sigin G	2/8/83	Beluran	Ulu Tungud FR
SAN85477	47	-	<LOD	-	*Glochidion*	*mindorense*	Arshid T	21/5/77	Sandakan	Sepilok Kabili FR
61552	49	-	<LOD	-	*Glochidion*	*mindorense*	Meijer W	17/8/67	Sandakan	Sepilok Kabili FR
SAN90786	<LOD	-	<LOD	-	*Glochidion*	*mindorense*	Madani L	11/7/79	Lahad Datu	Pulau Silumpat
SAN26683	<LOD	-	<LOD	-	*Glochidion*	*mindorense*	Chai M	17/10/61	Lahad Datu	Pulau Tabawan
SAN26683	<LOD	-	<LOD	-	*Glochidion*	*mindorense*	Chai M	17/10/61	Lahad Datu	Pulau Tabawan
SAN149384	<LOD	-	<LOD	-	*Glochidion*	*mindorense*	Pius G	23/10/07	Lahad Datu	Silam
SAN25052	<LOD	-	<LOD	-	*Glochidion*	*mindorense*	Chai M	1/6/61	Lahad Datu	Silam
56177	<LOD	-	<LOD	-	*Glochidion*	*mindorense*	Kanis A	16/5/66	Tawau	Tawau
rsnb4017	<LOD	-	<LOD	-	*Glochidion*	*monostylum*	Chew WL	20/1/64	Ranau	Mesilau
271	<LOD	-	<LOD	-	*Glochidion*	*monostylum*	Chew WL	5/8/61	Ranau	Mt.Kinabalu
35331	<LOD	-	60	-	*Glochidion*	*obscurum*	Jawanting A	28/5/63	Kinabatangan	Lamag
SAN71165	<LOD	-	79	-	*Glochidion*	*obscurum*	Diwol S		Beluran	Telupid
SAN114939	<LOD	-	<LOD	-	*Glochidion*	*obscurum*	Amin AAS	16/12/89	Beaufort	Beaufort
SAN127335	<LOD	-	<LOD	-	*Glochidion*	*obscurum*	Amin AAS	26/9/90	Beaufort	Beaufort
SAN84397	<LOD	-	<LOD	-	*Glochidion*	*obscurum*	Talib B	25/8/76	Beaufort	Beaufort
SAN113631	<LOD	-	<LOD	-	*Glochidion*	*obscurum*	Gambating A	29/10/85	Beluran	Beluran
SAN67130	<LOD	-	<LOD	-	*Glochidion*	*obscurum*	Aban G	17/9/84	Beluran	Beluran
SAN139644	<LOD	-	<LOD	-	*Glochidion*	*obscurum*	Sumbing J	22/1/95	Nabawan	Bkt Pisagan

SAN66807	<LOD	-	<LOD	-	*Glochidion*	*obscurum*	Aban G	22/4/70	Tenom	Crocker Range NP
SAN94881	<LOD	-	<LOD	-	*Glochidion*	*obscurum*	Fidilis K	12/6/82	Tawau	Kalabakan
SAN73283	<LOD	-	<LOD	-	*Glochidion*	*obscurum*	Saikeh L	21/5/71	Keningau	Keningau
SAN28799	<LOD	-	<LOD	-	*Glochidion*	*obscurum*	Lajangah JK	20/9/62	Papar	Kimanis
SAN80462	<LOD	-	<LOD	-	*Glochidion*	*obscurum*	Diwol S	20/3/76	Papar	Kimanis
SAN33958	<LOD	-	<LOD	-	*Glochidion*	*obscurum*	Ahwing J	16/1/63	Kinabatangan	Kretam FR
SAN81733	<LOD	-	<LOD	-	*Glochidion*	*obscurum*	Madani L	21/6/75	Kinabatangan	Kuala Karamuak
SAN136394	<LOD	-	<LOD	-	*Glochidion*	*obscurum*	Diwol S	11/8/03	Kinabatangan	Maliau Basin
SAN114663	<LOD	-	<LOD	-	*Glochidion*	*obscurum*	Amin AAS	9/7/87	Papar	Mandahan
SAN126237	<LOD	-	<LOD	-	*Glochidion*	*obscurum*	Amin AAS	18/8/90	Papar	Mandahan
SAN99351	<LOD	-	<LOD	-	*Glochidion*	*obscurum*	Diwol S	13/8/83	Ranau	Matopang
SAN99351	<LOD	-	<LOD	-	*Glochidion*	*obscurum*	Diwol S	13/8/83	Ranau	Matopang
65226	<LOD	-	<LOD	-	*Glochidion*	*obscurum*	Aban G	7/3/69	Sipitang	Mendolong
41381	<LOD	-	<LOD	-	*Glochidion*	*obscurum*	Corpuz FM	25/1/64	Papar	Papar
SAN60257	<LOD	-	<LOD	-	*Glochidion*	*obscurum*	Sigin G	29/8/84	Kinabatangan	Pinangah
SAN109357	<LOD	-	<LOD	-	*Glochidion*	*obscurum*	Gambating A	13/6/85	Ranau	Ranau
SAN109707	<LOD	-	<LOD	-	*Glochidion*	*obscurum*	Sigin G	17/6/85	Ranau	Ranau
SAN114250	<LOD	-	<LOD	-	*Glochidion*	*obscurum*	Gambating A	17/3/86	Ranau	Ranau
SAN11567	<LOD	-	<LOD	-	*Glochidion*	*obscurum*	Amin AAS	17/7/86	Ranau	Ranau
SAN116426	<LOD	-	<LOD	-	*Glochidion*	*obscurum*	Gambating A	16/10/86	Ranau	Ranau
SAN118115	<LOD	-	<LOD	-	*Glochidion*	*obscurum*	Gambating A	18/6/87	Ranau	Ranau
SAN128349	<LOD	-	<LOD	-	*Glochidion*	*obscurum*	Fidilis K	19/2/90	Nabawan	Sapulut
SAN16064	<LOD	-	<LOD	-	*Glochidion*	*obscurum*	Wood GHS	3/4/55	Lahad Datu	Segama
SAN25990	<LOD	-	<LOD	-	*Glochidion*	*obscurum*	Chai M	6/8/61	Lahad Datu	Segama
SAN26667	<LOD	-	<LOD	-	*Glochidion*	*obscurum*	Chai M	11/10/61	Lahad Datu	Segama
SAN115470	<LOD	-	<LOD	-	*Glochidion*	*obscurum*	Amin AAS	10/4/88	Sipitang	Sipitang
SAN123869	<LOD	-	<LOD	-	*Glochidion*	*obscurum*	Bousi J	27/4/88	Tambunan	Tambunan

SAN117200	<LOD	-	<LOD	-	*Glochidion*	*obscurum*	Maikin L	27/12/86	Kinabatangan	Tongod
SAN111533	<LOD	-	<LOD	-	*Glochidion*	*obscurum*	Gambating A	26/10/85	Beluran	Ulu Sapa Payau FR
SAN92095	<LOD	-	<LOD	-	*Glochidion*	*obscurum*	LasSAN P	6/5/80	Kinabatangan	Ulu Segama FR
63895	<LOD	-	<LOD	-	*Glochidion*	*obscurum*	Bongsu A	15/9/68	Tawau	Umas Umas
63894	<LOD	-	<LOD	-	*Glochidion*	*perakense*	Bongsu A	14/9/68	Tawau	Umas Umas
51062	84	-	<LOD	-	*Glochidion*	*philippicum*	Sinanggul HT	15/5/65	Kudat	Loro FR
38968	95	-	<LOD	-	*Glochidion*	*philippicum*	Ahwing J	30/7/63	Sandakan	Sandakan
SAN84663	<LOD	-	<LOD	-	*Glochidion*	*philippicum*	Talib B	23/7/77	Beaufort	Beaufort
SAN71603	<LOD	-	<LOD	-	*Glochidion*	*philippicum*	Jahudin B	18/2/71	Beluran	Beluran
50161	<LOD	-	<LOD	-	*Glochidion*	*philippicum*	Madani L	11/8/67	Sandakan	Garinono FR
SAN32788	<LOD	-	<LOD	-	*Glochidion*	*philippicum*	Jawanting A	30/1/63	Kinabatangan	Lamag
SAN74254	<LOD	-	<LOD	-	*Glochidion*	*philippicum*	Imbongan A	15/9/71	Kinabatangan	Lamag
SAN121165	<LOD	-	<LOD	-	*Glochidion*	*philippicum*	Gambating A	19/11/87	Ranau	Muruk
35065	<LOD	-	<LOD	-	*Glochidion*	*philippicum*	Madani L	21/3/63	Nabawan	Pensiangan
35065	<LOD	-	<LOD	-	*Glochidion*	*philippicum*	Madani L	21/3/63	Nabawan	Pensiangan
32868	<LOD	-	<LOD	-	*Glochidion*	*philippicum*	Jawanting A	22/3/63	Sandakan	Sandakan
SAN64511	<LOD	-	<LOD	-	*Glochidion*	*philippicum*	Madani L	10/7/68	Sandakan	Sepilok Kabili FR
SAN81132	58	-	<LOD	-	*Glochidion*	*pubicapsa*	Aban G	15/1/75	Beluran	Segaliud Lokan FR
SAN69253	<LOD	-	237	-	*Glochidion*	*pubicapsa*	Saikeh L	7/4/70	Tawau	Luasong
SAN80143	<LOD	-	<LOD	-	*Glochidion*	*pubicapsa*	Diwol S	12/8/75	Beaufort	Beaufort
SAN82269	<LOD	-	<LOD	-	*Glochidion*	*pubicapsa*	Aban G	10/9/75	Kinabatangan	Ganduman FR
SAN123810	<LOD	-	<LOD	-	*Glochidion*	*pubicapsa*	Bousi J	25/1/88	Kinabatangan	Gomantong FR
SAN75868	<LOD	-	<LOD	-	*Glochidion*	*pubicapsa*	Arshid T	26/9/72	Ranau	Kinabalu National Park
SAN31698	<LOD	-	<LOD	-	*Glochidion*	*pubicapsa*	Chai M	9/1/63	Lahad Datu	Lahad Datu
54781	<LOD	-	<LOD	-	*Glochidion*	*pubicapsa*	Talip AH	19/3/66	Lahad Datu	Lahad Datu
SAN29679	<LOD	-	<LOD	-	*Glochidion*	*pubicapsa*	Tahir M	20/5/62	Lahad Datu	Mostyn

SAN32988	<LOD	-	<LOD	-	*Glochidion*	*pubicapsa*	Aban G	11/12/62	Tawau	Quoin Hill FR
SAN81459	<LOD	-	<LOD	-	*Glochidion*	*pubicapsa*	Madani L	13/3/75	Beluran	Segaliud Lokan FR
SAN74594	<LOD	-	<LOD	-	*Glochidion*	*pubicapsa*	Diwol S	11/12/71	Beluran	Telupid
SAN124464	38	-	<LOD	-	*Glochidion*	*rubrum*	Madani L	23/4/88	Kinabatangan	Maliau Basin
SAN66945	42	-	<LOD	-	*Glochidion*	*rubrum*	Aban G	12/9/70	Beaufort	Beaufort
SAN116912	42	-	<LOD	-	*Glochidion*	*rubrum*	Bousi J	8/8/86	Kinabatangan	Ulu Segama FR
SAN75325	43	-	<LOD	-	*Glochidion*	*rubrum*	Kumin M	7/4/72	Tawau	Kalabakan
SAN30596	47	-	<LOD	-	*Glochidion*	*rubrum*	Aban G	21/7/62	Tawau	Kalabakan
SAN75956	66	-	<LOD	-	*Glochidion*	*rubrum*	Shea G	6/9/72	Pitas	Bengkoka FR
SAN97421	69	-	<LOD	-	*Glochidion*	*rubrum*	Gambating A	28/6/83	Kinabatangan	Tangkulap
SAN90422	112	-	<LOD	-	*Glochidion*	*rubrum*	Gambating A	27/5/82	Sandakan	Sepilok Kabili FR
SAN71618	133	-	<LOD	-	*Glochidion*	*rubrum*	Jahudin B	19/2/71	Beluran	Beluran
SAN118873	<LOD	-	1411	2537	*Glochidion*	*rubrum*	Gambating A	9/10/87	Ranau	Bukit Kolong
SAN24500	<LOD	-	<LOD	-	*Glochidion*	*rubrum*	Brand D	7/3/61	Tawau	Apas
SAN77497	<LOD	-	<LOD	-	*Glochidion*	*rubrum*	Diwol S	21/3/73	Penampang	Babagon
SAN72153	<LOD	-	<LOD	-	*Glochidion*	*rubrum*	Saikeh L	6/6/72	Beaufort	Beaufort
SAN80190	<LOD	-	<LOD	-	*Glochidion*	*rubrum*	Diwol S	23/8/75	Beaufort	Beaufort
SAN100347	<LOD	-	<LOD	-	*Glochidion*	*rubrum*	Amin AAS	15/9/84	Beluran	Beluran
SAN107630	<LOD	-	<LOD	-	*Glochidion*	*rubrum*	Ismail AAP	17/1/85	Beluran	Beluran
SAN97609	<LOD	-	<LOD	-	*Glochidion*	*rubrum*	Aban G	11/7/83	Beluran	Beluran
SAN59835	<LOD	-	<LOD	-	*Glochidion*	*rubrum*	Rahim A	14/11/83	Kinabatangan	Bilit
SAN84405	<LOD	-	<LOD	-	*Glochidion*	*rubrum*	Talib B	9/9/76	Papar	Bongawan
SAN81877	<LOD	-	<LOD	-	*Glochidion*	*rubrum*	Aban G	12/7/75	Beluran	Bonggaya FR
SAN78393	<LOD	-	<LOD	-	*Glochidion*	*rubrum*	Diwol S	20/9/74	Tenom	Crocker Range NP
SAN112931	<LOD	-	<LOD	-	*Glochidion*	*rubrum*	Madani L	25/11/85	Lahad Datu	Danum Valley
SAN114514	<LOD	-	<LOD	-	*Glochidion*	*rubrum*	Madani L	9/7/86	Lahad Datu	Danum Valley
SAN85323	<LOD	-	<LOD	-	*Glochidion*	*rubrum*	Stone BC	3/9/76	Lahad Datu	Danum Valley

SAN91408	<LOD	-	<LOD	-	*Glochidion*	*rubrum*	Fidilis K	14/12/79	Kinabatangan	Imbak Valley
SAN95691	<LOD	-	<LOD	-	*Glochidion*	*rubrum*	Fidilis K	24/2/83	Kinabatangan	Imbak Valley
SAN101434	<LOD	-	<LOD	-	*Glochidion*	*rubrum*	Fidilis K	24/11/83	Tawau	Kalabakan
SAN24631	<LOD	-	<LOD	-	*Glochidion*	*rubrum*	Burgess PF	22/2/61	Tawau	Kalabakan
SAN75671	<LOD	-	<LOD	-	*Glochidion*	*rubrum*	Chow J	15/4/72	Tawau	Kalabakan
SAN96054	<LOD	-	<LOD	-	*Glochidion*	*rubrum*	Fidilis K	23/3/83	Tawau	Kalabakan
SAN25951	<LOD	-	<LOD	-	*Glochidion*	*rubrum*	Chai M	24/7/61	Kunak	Kalumpang FR
41277	<LOD	-	<LOD	-	*Glochidion*	*rubrum*	Jawanting A	8/1/64	Papar	Kawang FR
SAN141439	<LOD	-	<LOD	-	*Glochidion*	*rubrum*	Meijer W	7/10/94	Sandakan	Kebun Cina
SAN60005	<LOD	-	<LOD	-	*Glochidion*	*rubrum*	Amin AAS	12/11/83	Keningau	Keningau
SAN70134	<LOD	-	<LOD	-	*Glochidion*	*rubrum*	Cockburn PF	29/9/72	Ranau	Kinabalu National Park
SAN33213	<LOD	-	<LOD	-	*Glochidion*	*rubrum*	Madani L	19/12/62	Kinabatangan	Kinabatangan
SAN79184	<LOD	-	<LOD	-	*Glochidion*	*rubrum*	-		Kinabatangan	Kuamut FR
SAN30960	<LOD	-	<LOD	-	*Glochidion*	*rubrum*	Brand D	24/7/63	Kudat	Kudat
44184	<LOD	-	<LOD	-	*Glochidion*	*rubrum*	Arshid T	15/7/64	Sandakan	Labuk Road FR
55483	<LOD	-	<LOD	-	*Glochidion*	*rubrum*	Aban G	19/5/66	Lahad Datu	Lahad Datu
SAN120963	<LOD	-	<LOD	-	*Glochidion*	*rubrum*	Majawat G	27/7/87	Kinabatangan	Lamag
SAN83241	<LOD	-	<LOD	-	*Glochidion*	*rubrum*	Cockburn PF	20/5/76	Kinabatangan	Lamag
36377	<LOD	-	<LOD	-	*Glochidion*	*rubrum*	Jawanting A	10/7/63	Kinabatangan	Lamag
SAN80601	<LOD	-	<LOD	-	*Glochidion*	*rubrum*	Talib B	23/6/76	Beaufort	Lingkungan
SAN36122	<LOD	-	<LOD	-	*Glochidion*	*rubrum*	Lajangah JK	18/5/63	Kudat	Loro FR
SAN68793	<LOD	-	<LOD	-	*Glochidion*	*rubrum*	Fidilis K	21/1/84	Tawau	Luasong
SAN82183	<LOD	-	<LOD	-	*Glochidion*	*rubrum*	Fidilis K	8/3/77	Tawau	Luasong
SAN89725	<LOD	-	<LOD	-	*Glochidion*	*rubrum*	Fidilis K	20/2/79	Tawau	Luasong
SAN77922	<LOD	-	<LOD	-	*Glochidion*	*rubrum*	Diwol S	10/9/73	Beaufort	Lumat
SAN27045	<LOD	-	<LOD	-	*Glochidion*	*rubrum*	Damit AA	25/9/61	Kunak	Madai Baturong FR

SAN99983	<LOD	-	<LOD	-	*Glochidion*	*rubrum*	Aban G	16/8/83	KotaMarudu	Marak Parak
SAN86529	<LOD	-	<LOD	-	*Glochidion*	*rubrum*	Amin AAS	17/7/85	Sipitang	Marintaman
SAN78116	<LOD	-	<LOD	-	*Glochidion*	*rubrum*	Diwol S	3/5/74	Nabawan	Nabawan
SAN85766	<LOD	-	<LOD	-	*Glochidion*	*rubrum*	Nordin AA	8/7/77	Nabawan	Nabawan
SAN94937	<LOD	-	<LOD	-	*Glochidion*	*rubrum*	Diwol S	9/3/82	Nabawan	Nabawan
SAN22249	<LOD	-	<LOD	-	*Glochidion*	*rubrum*	Charington C	2/7/60	Tenom	Pangi
SAN67494	<LOD	-	<LOD	-	*Glochidion*	*rubrum*	Diwol S	11/2/85	Kinabatangan	Pinangah
SAN86769	<LOD	-	<LOD	-	*Glochidion*	*rubrum*	Stone BC	11/4/77	Kudat	Pulau Balambangan
SAN26654	<LOD	-	<LOD	-	*Glochidion*	*rubrum*	Chai M	9/10/61	Lahad Datu	Pulau Sakar
SAN19626	<LOD	-	<LOD	-	*Glochidion*	*rubrum*	Meijer W	18/7/59	Tawau	Pulau Sebatik
SAN116242	<LOD	-	<LOD	-	*Glochidion*	*rubrum*	Gambating A	24/9/86	Ranau	Ranau
SAN117205	<LOD	-	<LOD	-	*Glochidion*	*rubrum*	Gambating A	21/11/86	Ranau	Ranau
SAN118122	<LOD	-	<LOD	-	*Glochidion*	*rubrum*	Gambating A	20/6/87	Ranau	Ranau
SAN121437	<LOD	-	<LOD	-	*Glochidion*	*rubrum*	Gambating A	16/11/87	Ranau	Ranau
SAN78610	<LOD	-	<LOD	-	*Glochidion*	*rubrum*	Madani L	16/7/73	Sandakan	Sandakan
35419	<LOD	-	<LOD	-	*Glochidion*	*rubrum*	Sayu E	14/3/63	Sandakan	Sandakan
SAN22199	<LOD	-	<LOD	-	*Glochidion*	*rubrum*	Burgess PF	4/10/60	Sandakan	Sapagaya FR
SAN81355	<LOD	-	<LOD	-	*Glochidion*	*rubrum*	Madani L	5/3/75	Beluran	Sapi
SAN106936	<LOD	-	<LOD	-	*Glochidion*	*rubrum*	Sumbing J	13/10/84	Nabawan	Sapulut
SAN124242	<LOD	-	<LOD	-	*Glochidion*	*rubrum*	Kulip J	12/7/88	Beluran	Segaliud Lokan FR
SAN140483	<LOD	-	<LOD	-	*Glochidion*	*rubrum*	Nasrah Y	31/10/95	Beluran	Segaliud Lokan FR
SAN81576	<LOD	-	<LOD	-	*Glochidion*	*rubrum*	Madani L	26/4/75	Beluran	Segaliud Lokan FR
33979	<LOD	-	<LOD	-	*Glochidion*	*rubrum*	Ahwing J	19/2/63	Beluran	Segaliud Lokan FR
SAN21285	<LOD	-	<LOD	-	*Glochidion*	*rubrum*	Meijer W	26/4/60	Sandakan	Sepilok Kabili FR
53488	<LOD	-	<LOD	-	*Glochidion*	*rubrum*	Kanis A	3/9/65	Sandakan	Sepilok Kabili FR
30978	<LOD	-	<LOD	-	*Glochidion*	*rubrum*	Brand D	15/9/62	Sandakan	Sepilok Kabili FR
SAN31171	<LOD	-	<LOD	-	*Glochidion*	*rubrum*	Aban G	25/8/62	Tawau	Serudong

jtp735	<LOD	-	<LOD	-	*Glochidion*	*rubrum*	Pereira JT	8/6/00	Kinabatangan	Sg Imbak FR
SAN66547	<LOD	-	<LOD	-	*Glochidion*	*rubrum*	Sigin G	22/8/84	Kinabatangan	Sg Pinanga FR
SAN80621	<LOD	-	<LOD	-	*Glochidion*	*rubrum*	Talib B	25/6/76	Beaufort	SiaungauFR
52808	<LOD	-	<LOD	-	*Glochidion*	*rubrum*	Talip AH	20/8/65	Lahad Datu	Silam
SAN114815	<LOD	-	<LOD	-	*Glochidion*	*rubrum*	Amin AAS	21/10/89	Sipitang	Sipitang
SAN82536	<LOD	-	<LOD	-	*Glochidion*	*rubrum*	Cockburn PF	19/10/75	Beluran	Sugut FR
34990	<LOD	-	<LOD	-	*Glochidion*	*rubrum*	Ahwing J	28/3/63	Lahad Datu	Tabin FR
SAN135278	<LOD	-	<LOD	-	*Glochidion*	*rubrum*	Diwol S	14/6/97	Lahad Datu	Tabin FR
SAN141952	<LOD	-	<LOD	-	*Glochidion*	*rubrum*	Kulip J	21/6/97	Lahad Datu	Tabin FR
37651	<LOD	-	<LOD	-	*Glochidion*	*rubrum*	Battah M	15/10/63	KotaMarudu	Tagaroh FR
SAN83644	<LOD	-	<LOD	-	*Glochidion*	*rubrum*	Kodoh T	16/7/76	Beluran	Telupid
SAN74603	<LOD	-	<LOD	-	*Glochidion*	*rubrum*	Shea G	25/10/71	Keningau	Tulid
SAN116912	<LOD	-	<LOD	-	*Glochidion*	*rubrum*	Bousi J	8/8/86	Kinabatangan	Ulu Segama FR
SAN71052	<LOD	-	<LOD	-	*Glochidion*	*rubrum*	Talip AH	20/7/70	Kinabatangan	Ulu Segama FR
SAN79132	<LOD	-	<LOD	-	*Glochidion*	*rubrum*	Free M	16/8/75	Kinabatangan	Ulu Segama FR
SAN84971	<LOD	-	<LOD	-	*Glochidion*	*rubrum*	Cockburn PF	19/8/76	Kinabatangan	UluSegama FR
SAN116429	51	-	<LOD	-	*Glochidion*	*sericeum*	Gambating A	16/10/86	Ranau	Ranau
56247	51	-	<LOD	-	*Glochidion*	*sericeum*	Sinanggul HT	4/6/66	Lahad Datu	Mostyn
SAN74953	<LOD	-	<LOD	-	*Glochidion*	*sericeum*	Diwol S	11/12/71	Beluran	Beluran
SAN129792	<LOD	-	<LOD	-	*Glochidion*	*sericeum*	Fidilis K	26/10/90	Keningau	Keningau
SAN85837	<LOD	-	<LOD	-	*Glochidion*	*sericeum*	Nordin AA	26/6/77	Keningau	Keningau
SAN80510	<LOD	-	<LOD	-	*Glochidion*	*sericeum*	Diwol S	26/3/76	Papar	Kimanis
SAN129552	<LOD	-	<LOD	-	*Glochidion*	*sericeum*	Diwol S	18/1/90	Lahad Datu	Lahad Datu
41621	<LOD	-	<LOD	-	*Glochidion*	*sericeum*	Agam A	16/4/64	Lahad Datu	Lahad Datu
SAN90696	<LOD	-	<LOD	-	*Glochidion*	*sericeum*	Aban G	12/11/79	Kinabatangan	Lamag
SAN95636	<LOD	-	<LOD	-	*Glochidion*	*sericeum*	Fidilis K	18/1/83	Tawau	Luasong
SAN66866	<LOD	-	<LOD	-	*Glochidion*	*sericeum*	Aban G	18/7/70	Beaufort	Membakut

SAN127982	<LOD	-	<LOD	-	*Glochidion*	*sericeum*	Asik M	18/9/89	Nabawan	Nabawan
SAN119038	<LOD	-	<LOD	-	*Glochidion*	*sericeum*	Bousi J	23/4/87	Kinabatangan	Pinangah
SAN26731	<LOD	-	<LOD	-	*Glochidion*	*sericeum*	Mujin MA	20/10/61	Ranau	Poring
SAN155555	<LOD	-	<LOD	-	*Glochidion*	*sericeum*	Alviana D	12/9/13	Tongod	Sg Imbak VJR
SAN33429	<LOD	-	<LOD	-	*Glochidion*	*sericeum*	Sitiol A	18/2/63	Lahad Datu	Silabukan FR
SAN78365	<LOD	-	<LOD	-	*Glochidion*	*sericeum*	Diwol S	17/7/74	Sipitang	Sipitang
SAN110414	<LOD	-	<LOD	-	*Glochidion*	*sericeum*	Gambating A	17/7/85	Ranau	Trus Madi FR
SAN95563	<LOD	-	<LOD	-	*Glochidion*	*sericeum*	Fidilis K	13/12/82	Kinabatangan	UluSegama FR
SAN30586	59	-	<LOD	-	*Glochidion*	*singaporense*	Aban G	29/7/62	Tawau	Kalabakan
SAN76078	<LOD	-	<LOD	-	*Glochidion*	*singaporense*	Shea G	9/9/72	Pitas	Bengkoka FR
SAN95599	46	-	<LOD	-	*Glochidion*	sp. un det.	Fidilis K	18/12/82	Kinabatangan	Ulu Segama FR
SAN144061	47	-	<LOD	-	*Glochidion*	sp. un det.	Postar JM	18/7/01	Kinabatangan	Maliau Basin
SAN113379	49	-	<LOD	-	*Glochidion*	sp. un det.	Asik M	18/2/86	Nabawan	Pandewan
SAN134502	52	-	<LOD	-	*Glochidion*	sp. un det.	Berhaman A	19/4/92	Tawau	Tawau Hill Park FR
fri41252	59	-	8037	9650	*Glochidion*	sp. un det.	Soepadmo E	4/4/94	Kinabatangan	Maliau Basin
SAN133343	59	-	3674	4967	*Glochidion*	sp. un det.	Kulip J	23/2/92	Lahad Datu	Mt Nicola
SAN117806	60	-	<LOD	-	*Glochidion*	sp. un det.	Gambating A	10/6/87	Ranau	Ranau
SAN118035	60	-	<LOD	-	*Glochidion*	sp. un det.	Kessler P	24/1/89	Sandakan	Sepilok Kabili FR
SAN130133	64	-	<LOD	-	*Glochidion*	sp. un det.	Sumbing J	14/7/92	Nabawan	Pensiangan FR
SAN137086	64	-	6779	8300	*Glochidion*	sp. un det.	Kulip J	2/3/93	Beluran	Telupid
SAN113912	70	-	68	-	*Glochidion*	sp. un det.	Asik M	25/3/86	Nabawan	Sapulut
SAN116337	71	-	<LOD	-	*Glochidion*	sp. un det.	Gambating A	9/10/86	Ranau	Mamut Copper Mine
fri74324	76	-	59	-	*Glochidion*	sp. un det.	Yao TL	4/12/10	Kinabatangan	ImbakV alley
SAN123998	97	-	<LOD	-	*Glochidion*	sp. un det.	Bousi J	4/5/88	Keningau	Trus Madi FR
SAN123593	163	-	265	-	*Glochidion*	sp. un det.	Gambating A	6/10/88	Ranau	Ranau
SAN72781	267	256	46	-	*Glochidion*	sp. un det.	Madani L	12/3/71	Beluran	Segaliud Lokan FR
SAN137049	310	270	4784	6158	*Glochidion*	sp. un det.	Kulip J	29/2/92	Kinabatangan	Bkt Tinkar

SAN96683	1527	681	4461	5811	*Glochidion*	sp. un det.	Diwol S	10/6/83	Kinabatangan	Bkt Tinkar
52431	<LOD	-	47	-	*Glochidion*	sp. un det.	Talip AH	21/5/65	Lahad Datu	Gn Silam
SAN139495	<LOD	-	94	-	*Glochidion*	sp. un det.	Sugau JB	10/8/04	Beluran	Ulu Tungud FR
SAN123105	<LOD	-	129	-	*Glochidion*	sp. un det.	Gambating A	1/4/88	Ranau	Ranau
SAN98107	<LOD	-	163	-	*Glochidion*	sp. un det.	Proctor J	17/8/83	Lahad Datu	Gn Silam
SAN56832	<LOD	-	2064	3238	*Glochidion*	sp. un det.	Sigin G	29/3/84	Beluran	Telupid
SAN25065	<LOD	-	5480	6905	*Glochidion*	sp. un det.	Chai M	3/6/61	Lahad Datu	Pulau Sakar
SAN94016	<LOD	-	5957	7418	*Glochidion*	sp. un det.	Aban G	5/8/81	Beluran	Telupid
36885	<LOD	-	<LOD	-	*Glochidion*	sp. un det.	Madani L	6/11/63	Beaufort	Beaufort
jtp2	<LOD	-	<LOD	-	*Glochidion*	sp. un det.	Pereira JT	28/6/94	Ranau	Bundu Tuhan
SAN152230	<LOD	-	<LOD	-	*Glochidion*	sp. un det.	Markus G	30/7/10	Keningau	Crocker Range
SAN131115	<LOD	-	<LOD	-	*Glochidion*	sp. un det.	Francis A	27/8/90	Lahad Datu	Danum Valley
SAN148042	<LOD	-	<LOD	-	*Glochidion*	sp. un det.	Julia S	5/7/06	Lahad Datu	Danum Valley
SAN92792	<LOD	-	<LOD	-	*Glochidion*	sp. un det.	Aban G	24/2/82	Sandakan	Garinono FR
SAN78456	<LOD	-	<LOD	-	*Glochidion*	sp. un det.	Diwol S	6/3/75	Beaufort	Gn Lumaku FR
jbs22	<LOD	-	<LOD	-	*Glochidion*	sp. un det.	Sugau JB	9/7/94	Lahad Datu	Gn Silam
SAN102071	<LOD	-	<LOD	-	*Glochidion*	sp. un det.	Lee YF	29/8/84	Lahad Datu	Gn Silam
SAN81292	<LOD	-	<LOD	-	*Glochidion*	sp. un det.	Arshid T	19/2/75	Sandakan	Gum-Gum FR
SAN77579	<LOD	-	<LOD	-	*Glochidion*	sp. un det.	Diwol S	9/5/73	Beaufort	Haligolat
SAN152695	<LOD	-	<LOD	-	*Glochidion*	sp. un det.	Suzana S	30/11/10	Kinabatangan	Imbak Canyon
fri74280	<LOD	-	<LOD	-	*Glochidion*	sp. un det.	Yao TL	30/11/10	Kinabatangan	Imbak Valley
SAN94781	<LOD	-	<LOD	-	*Glochidion*	sp. un det.	Fidilis K	22/4/82	Tawau	Kalabakan
41366	<LOD	-	<LOD	-	*Glochidion*	sp. un det.	Jawanting A	9/2/64	KotaBelud	Kelawat FR
SAN110906	<LOD	-	<LOD	-	*Glochidion*	sp. un det.	Asik M	13/3/85	Keningau	Keningau
SAN87660	<LOD	-	<LOD	-	*Glochidion*	sp. un det.	Aban G	20/7/78	Ranau	Kinabalu National Park
SAN68082	<LOD	-	<LOD	-	*Glochidion*	sp. un det.	Saikeh L	14/10/69	Kunak	Kunak

SAN25246	<LOD	-	<LOD	-	*Glochidion*	sp. un det.	Mujin MA	8/8/61	Ranau	Kundasang
SAN71372	<LOD	-	<LOD	-	*Glochidion*	sp. un det.	Talip AH	25/11/70	Sandakan	Labuk Road FR
35579	<LOD	-	<LOD	-	*Glochidion*	sp. un det.	Sayu E	25/3/63	Sandakan	Labuk Road FR
SAN31623	<LOD	-	<LOD	-	*Glochidion*	sp. un det.	Chai M	11/10/62	Lahad Datu	Lahad Datu
SAN52028	<LOD	-	<LOD	-	*Glochidion*	sp. un det.	Banang E	12/10/65	Kinabatangan	Lamag
62162	<LOD	-	<LOD	-	*Glochidion*	sp. un det.	Jumin	4/6/68	Kinabatangan	Lamag
52016	<LOD	-	<LOD	-	*Glochidion*	sp. un det.	Banang E	12/10/65	Kinabatangan	Lamag
SAN118440	<LOD	-	<LOD	-	*Glochidion*	sp. un det.	Asik M	20/10/86	Keningau	Lanas
SAN118440	<LOD	-	<LOD	-	*Glochidion*	sp. un det.	Asik M	20/10/86	Keningau	Lanas
SAN149893	<LOD	-	<LOD	-	*Glochidion*	sp. un det.	Joel D	24/11/08	Beluran	Lipaso FR
SAN143211	<LOD	-	<LOD	-	*Glochidion*	sp. un det.	Lim SP	12/4/00	Sipitang	Long Miau
462	<LOD	-	<LOD	-	*Glochidion*	sp. un det.	Geofarry G	29/6/97	Sipitang	Long Pasia
SAN23638	<LOD	-	<LOD	-	*Glochidion*	sp. un det.	Meijer W	11/11/60	Beluran	Lubok Buaya
SAN140188	<LOD	-	<LOD	-	*Glochidion*	sp. un det.	Nasrah Y	26/8/95	Beluran	Lung Manis
SAN16274	<LOD	-	<LOD	-	*Glochidion*	sp. un det.	Wood GHS	17/9/55	Sipitang	Malaman
SAN144062	<LOD	-	<LOD	-	*Glochidion*	sp. un det.	Postar JM	18/7/01	Kinabatangan	Maliau Basin
23924	<LOD	-	<LOD	-	*Glochidion*	sp. un det.	Sario IH	25/6/64	Beluran	Mamahat FR
SAN86249	<LOD	-	<LOD	-	*Glochidion*	sp. un det.	Madani L	4/11/84	Sipitang	Marintaman
SAN86249	<LOD	-	<LOD	-	*Glochidion*	sp. un det.	Madani L	4/11/84	Sipitang	Marintaman
SAN103279	<LOD	-	<LOD	-	*Glochidion*	sp. un det.	Amin AAS	21/11/76	Beaufort	Membakut
SAN84748	<LOD	-	<LOD	-	*Glochidion*	sp. un det.	Talib B	14/11/78	Beaufort	Membakut
SAN72292	<LOD	-	<LOD	-	*Glochidion*	sp. un det.	Saikeh L	12/10/72	Sipitang	Mendolong
SAN140567	<LOD	-	<LOD	-	*Glochidion*	sp. un det.	Maidil A	10/4/96	Sipitang	Mesapol
SAN133585	<LOD	-	<LOD	-	*Glochidion*	sp. un det.	Kulip J	23/4/97	Sipitang	Mesapol FR
SAN77838	<LOD	-	<LOD	-	*Glochidion*	sp. un det.	Diwol S	17/8/73	Sipitang	Mesapol FR
SAN123518	<LOD	-	<LOD	-	*Glochidion*	sp. un det.	Gambating A	2/9/88	Ranau	Mesilau
SAN149457	<LOD	-	<LOD	-	*Glochidion*	sp. un det.	Suzana S	13/5/08	Semporna	Mt Pock FR

jtp837	<LOD	-	<LOD	-	*Glochidion*	sp. un det.	Pereira JT	1/9/01	Nabawan	Nabawan
SAN104333	<LOD	-	<LOD	-	*Glochidion*	sp. un det.	Fidilis K	19/5/87	Nabawan	Nabawan
SAN128056	<LOD	-	<LOD	-	*Glochidion*	sp. un det.	Sumbing J	12/3/90	Nabawan	Nabawan
SAN80874	<LOD	-	<LOD	-	*Glochidion*	sp. un det.	Diwol S	16/6/76	Nabawan	Nabawan
SAN121518	<LOD	-	<LOD	-	*Glochidion*	sp. un det.	Gambating A	28/4/88	Ranau	Nalapak
SAN101933	<LOD	-	<LOD	-	*Glochidion*	sp. un det.	Soinin S	16/10/89	Tuaran	Pahu
SAN121353	<LOD	-	<LOD	-	*Glochidion*	sp. un det.	Pius G	20/5/87	Pitas	Paitan FR
51643	<LOD	-	<LOD	-	*Glochidion*	sp. un det.	Meijer W	20/5/65	Beluran	Pamol
SAN141798	<LOD	-	<LOD	-	*Glochidion*	sp. un det.	Diwol S	22/3/99	Tenom	Pangi
SAN22208	<LOD	-	<LOD	-	*Glochidion*	sp. un det.	Charington C	30/6/60	Tenom	Pangi
SAN42295	<LOD	-	<LOD	-	*Glochidion*	sp. un det.	Jawanting A	20/5/64	Tenom	Pangi
SAN42315	<LOD	-	<LOD	-	*Glochidion*	sp. un det.	Jawanting A	17/5/64	Tenom	Pangi
SAN129338	<LOD	-	<LOD	-	*Glochidion*	sp. un det.	Gambating A	20/10/88	Ranau	Poring
sip-b24	<LOD	-	<LOD	-	*Glochidion*	sp. un det.	Sugau JB	11/10/98	Semporna	Pulau Boheydulang
SAN109716	<LOD	-	<LOD	-	*Glochidion*	sp. un det.	Sigin G	17/6/85	Ranau	Ranau
SAN116343	<LOD	-	<LOD	-	*Glochidion*	sp. un det.	Gambating A	10/10/86	Ranau	Ranau
SAN117806	<LOD	-	<LOD	-	*Glochidion*	sp. un det.	Gambating A	10/6/87	Ranau	Ranau
SAN117943	<LOD	-	<LOD	-	*Glochidion*	sp. un det.	Gambating A	16/6/87	Ranau	Ranau
SAN123119	<LOD	-	<LOD	-	*Glochidion*	sp. un det.	Gambating A	4/4/88	Ranau	Ranau
SAN129390	<LOD	-	<LOD	-	*Glochidion*	sp. un det.	Gambating A	5/1/89	Ranau	Ranau
SAN137115	<LOD	-	<LOD	-	*Glochidion*	sp. un det.	Kulip J	4/2/93	Ranau	Ranau
SAN141702	<LOD	-	<LOD	-	*Glochidion*	sp. un det.	Meijer W	26/7/94	Ranau	Ranau
SAN76915	<LOD	-	<LOD	-	*Glochidion*	sp. un det.	Shea G	11/5/73	Ranau	Ranau
57696	<LOD	-	<LOD	-	*Glochidion*	sp. un det.	Meijer W		Sandakan	Sandakan
SAN135752	<LOD	-	<LOD	-	*Glochidion*	sp. un det.	Fidilis K	28/1/93	Nabawan	Sapulut
SAN60812	<LOD	-	<LOD	-	*Glochidion*	sp. un det.	Fox JED	7/8/70	Beluran	Segaliud Lokan FR
SAN73632	<LOD	-	<LOD	-	*Glochidion*	sp. un det.	Madani L	16/6/71	Beluran	Segaliud Lokan FR

SAN73633	<LOD	-	<LOD	-	*Glochidion*	sp. un det.	Madani L	16/6/71	Beluran	Segaliud Lokan FR
SAN73637	<LOD	-	<LOD	-	*Glochidion*	sp. un det.	Madani L	17/6/71	Beluran	Segaliud Lokan FR
SAN73931	<LOD	-	<LOD	-	*Glochidion*	sp. un det.	Madani L	23/6/71	Beluran	Segaliud Lokan FR
3017	<LOD	-	<LOD	-	*Glochidion*	sp. un det.	Jumaat A	21/9/83	Sandakan	Sepilok Kabili FR
SAN24445	<LOD	-	<LOD	-	*Glochidion*	sp. un det.	Charington C	3/3/61	Sandakan	Sepilok Kabili FR
SAN73084	<LOD	-	<LOD	-	*Glochidion*	sp. un det.	Talip AH	24/6/76	Sandakan	Sepilok Kabili FR
57478	<LOD	-	<LOD	-	*Glochidion*	sp. un det.	Sam PP	20/10/66	Sandakan	Sepilok Kabili FR
61636	<LOD	-	<LOD	-	*Glochidion*	sp. un det.	Binideh RP	22/8/67	Sandakan	Sepilok Kabili FR
34273	<LOD	-	<LOD	-	*Glochidion*	sp. un det.	Meijer W	15/10/63	Sandakan	Sepilok Kabili FR
58003	<LOD	-	<LOD	-	*Glochidion*	sp. un det.	Sinanggul HT	17/4/67	Lahad Datu	Silabukan FR
SAN149377	<LOD	-	<LOD	-	*Glochidion*	sp. un det.	Pius G	23/10/07	Lahad Datu	Silam
SAN138903	<LOD	-	<LOD	-	*Glochidion*	sp. un det.	Ignatius B	12/8/94	Sipitang	Sipitang
SAN138903	<LOD	-	<LOD	-	*Glochidion*	sp. un det.	Ignatius B	12/8/94	Sipitang	Sipitang
SAN139696	<LOD	-	<LOD	-	*Glochidion*	sp. un det.	Ignatius B	27/7/04	Sipitang	Sipitang
SAN141764	<LOD	-	<LOD	-	*Glochidion*	sp. un det.	Diwol S	21/3/99	Sipitang	Sipitang
SAN143183	<LOD	-	<LOD	-	*Glochidion*	sp. un det.	Pius G	14/4/00	Sipitang	Sipitang
SAN150503	<LOD	-	<LOD	-	*Glochidion*	sp. un det.	Suzana S	2/7/09	Beluran	Taviu FR
SAN142577	<LOD	-	<LOD	-	*Glochidion*	sp. un det.	Goh SH	15/6/98	Tawau	Tawau
SAN109475	<LOD	-	<LOD	-	*Glochidion*	sp. un det.	Lideh S	11/10/85	Beluran	Telupid
SAN120138	<LOD	-	<LOD	-	*Glochidion*	sp. un det.	Fidilis K	14/3/87	Tenom	Tenom
SAN120138	<LOD	-	<LOD	-	*Glochidion*	sp. un det.	Fidilis K	14/3/87	Tenom	Tenom
SAN130877	<LOD	-	<LOD	-	*Glochidion*	sp. un det.	?	17/5/90	Tenom	Tomani
SAN122195	<LOD	-	<LOD	-	*Glochidion*	sp. un det.	Fidilis K	26/9/88	Keningau	Tulid
SAN151909	<LOD	-	<LOD	-	*Glochidion*	sp. un det.	Suzana S	19/5/10	Keningau	Ulu Senagang
SAN146141	<LOD	-	<LOD	-	*Glochidion*	sp. un det.	Saw LG	23/7/05	Beluran	Ulu Tungud FR
SAN146658	<LOD	-	<LOD	-	*Glochidion*	sp. un det.	Saw LG	24/7/05	Beluran	Ulu Tungud FR
SAN146809	<LOD	-	<LOD	-	*Glochidion*	sp. un det.	Julia S	23/7/05	Beluran	Ulu Tungud FR

SAN86353	<LOD	-	<LOD	-	*Glochidion*	sp. un det.	Amin AAS	16/4/85	Beaufort	Weston
SAN150348	146	-	7063	8605	*Glochidion*	sp.1	Suzana S	20/3/09	Beluran	Bidu-Bidu FR
SAN150455	346	282	3434	4709	*Glochidion*	sp.2	Suzana S	24/3/09	Beluran	Bidu-Bidu FR
SAN150400	418	307	5748	7193	*Glochidion*	sp.2	Suzana S	24/3/09	Beluran	Bidu-Bidu FR
53269	49	-	<LOD	-	*Glochidion*	*superbum*	Kodoh T	18/6/65	Kinabatangan	Kuala Karamuak
57691	<LOD	-	<LOD	-	*Glochidion*	*superbum*	Zain M	26/4/67	Sandakan	Batu Sapi
41119	<LOD	-	<LOD	-	*Glochidion*	*superbum*	Meijer W	1963	Sandakan	Kebun Cina
SAN84040	<LOD	-	<LOD	-	*Glochidion*	*superbum*	Diwol S	23/11/76	Keningau	Keningau
SAN102962	<LOD	-	<LOD	-	*Glochidion*	*superbum*	Amin AAS	18/11/85	Beaufort	Kg Hindian FR
SAN73586	<LOD	-	<LOD	-	*Glochidion*	*superbum*	Kumin M	12/8/71	Sandakan	Leila FR
47348	<LOD	-	<LOD	-	*Glochidion*	*superbum*	Ampon B	4/5/65	Sandakan	Leila FR
37598	<LOD	-	<LOD	-	*Glochidion*	*superbum*	Hashim A	11/9/63	Sandakan	Leila FR
SAN80292	<LOD	-	<LOD	-	*Glochidion*	*superbum*	Karim AM	8/11/75	Beaufort	Maraba
SAN139199	<LOD	-	<LOD	-	*Glochidion*	*superbum*	Sumbing J	7/7/95	Tenom	Melalap
SAN99704	<LOD	-	<LOD	-	*Glochidion*	*superbum*	Sigin G	26/7/83	Beluran	Sg Meliau
SAN155374	<LOD	-	<LOD	-	*Glochidion*	*superbum*	Alviana D	11/9/13	Tongod	Sg ImbakVJR
SAN72484	<LOD	-	<LOD	-	*Glochidion*	*superbum*	Maurus	5/12/72	Keningau	Sook
SAN92425	<LOD	-	<LOD	-	*Glochidion*	*superbum*	Diwol S	24/7/80	Beluran	Tangkunan
SAN84340	<LOD	-	<LOD	-	*Glochidion*	*superbum*	Nordin AA	17/3/77	Keningau	Tulid
SAN85607	<LOD	-	<LOD	-	*Glochidion*	*superbum*	Nordin AA	14/4/77	Keningau	Tulid
SAN85830	<LOD	-	<LOD	-	*Glochidion*	*superbum*	Nordin AA	26/7/77	Keningau	Tulid
fri36228	43	-	<LOD	-	*Glochidion*	*wallichianum*	Saw LG	27/4/88	Kinabatangan	Maliau Basin
SAN120515	<LOD	-	<LOD	-	*Glochidion*	*wallichianum*	Majawat G	16/7/87	Kinabatangan	Dagat FR
SAN119481	<LOD	-	<LOD	-	*Glochidion*	*wallichianum*	Sawan T	18/6/87	Keningau	Keningau
SAN116756	<LOD	-	<LOD	-	*Glochidion*	*wallichianum*	Suin G	18/6/86	Beluran	Lung Manis
SAN35067	<LOD	-	<LOD	-	*Glochidion*	*wallichianum*	Madani L	21/3/63	Nabawan	Pensiangan
